# *Sp8 *exhibits reciprocal induction with *Fgf8 *but has an opposing effect on anterior-posterior cortical area patterning

**DOI:** 10.1186/1749-8104-2-10

**Published:** 2007-05-17

**Authors:** Setsuko Sahara, Yasuhiko Kawakami, Juan Carlos Izpisua Belmonte, Dennis DM O'Leary

**Affiliations:** 1 Molecular Neurobiology Laboratory, The Salk Institute, N. Torrey Pines Road, La Jolla, CA 92037, USA; 2 Gene Expression Laboratory, The Salk Institute, N. Torrey Pines Road, La Jolla, CA 92037, USA

## Abstract

Telencephalic patterning centers, defined by the discrete expression domains of distinct morphogens, *Fgf*s in the commissural plate (CoP), *Wnt*s and *Bmp*s in the cortical hem, and a ventral domain of Sonic hedgehog (*Shh*), are postulated to establish during development the initial patterning of the telencepahlon, including the neocortex. We show that the expression patterns of *Sp5*, *Sp8*, and *Sp9*, members of the *Sp8*-like family that are homologues of *Drosophila buttonhead*, correlate during early embryonic development with these three telencephalic patterning centers. To study potential functional relationships, we focused on *Sp8*, because it is transiently expressed in the CoP coincident with the expression of *Fgf8*, a morphogen implicated in area patterning of the neocortex. We also show that *Sp8 *is expressed in cortical progenitors in a high to low anterior-medial to posterior-lateral gradient across the ventricular zone. We used *in utero *electroporation of full-length and chimeric expression constructs to perform gain-of-function and loss-of-function studies of interactions between *Sp8 *and *Fgf8 *and their roles in cortical area patterning. We show that *Fgf8 *and *Sp8 *exhibit reciprocal induction *in vivo *in the embryonic telencephalon. *Sp8 *also induces downstream targets of *Fgf8*, including ETS transcription factors. *In vitro *assays show that Sp8 binds *Fgf8 *regulatory elements and is a direct transcriptional activator of *Fgf8*. We also show that Sp8 induction of *Fgf8 *is repressed by Emx2 *in vitro*, suggesting a mechanism to limit *Fgf8 *expression to the CoP. *In vivo *expression of a dominant negative *Sp8 *in the CoP indicates that *Sp8 *maintains expression of *Fgf8 *and also its effect on area patterning. Ectopic expression of Sp8 in anterior or posterior cortical poles induces significant anterior or posterior shifts in area patterning, respectively, paralleled by changes in expression of gene markers of positional identity. These effects of *Sp8 *on area patterning oppose those induced by ectopic expression of *Fgf8*, suggesting that in parallel to regulating *Fgf8 *expression, *Sp8 *also activates a distinct signaling pathway for cortical area patterning. In summary, *Sp8 *and *Fgf8 *robustly induce one another, and may act to balance the anterior-posterior area patterning of the cortex.

## Background

Many homeotic genes originally identified in *Drosophila *have important roles in vertebrate development. *Buttonhead *(*btd*), *empty spiracles *(*ems*), and *orthodenticles *(*otd*) were identified through mutagenesis screens as genes required for anterior head development in *Drosophila *[1-3]. Vertebrate homologues of *ems *and *otd*, which encode the Emx and Otx families of transcription factors (TFs), respectively, have been studied extensively, particularly their roles in mammalian forebrain development [4,5]. Vertebrate homologues of *btd*, a member of the *Sp1*-Zn finger family of TFs, have also been identified [6-8], but in contrast to homologues of *ems *and *otd*, they have been studied relatively little, particularly their roles in brain development.

Nine genes have been identified in mammals as *btd *homologues and the proteins they encode share *btd *and Zn finger domains. Based on the domain structures of their protein products, these nine genes are divided into a *Sp1*-like family (*Sp1-4*) and a *Sp8*-like family (*Sp5–9*). However, because *Sp8 *can rescue the defects in head development in *Drosophila btd *mutants, whereas *Sp1 *cannot [9], *Sp8 *and its family are viewed as the functional homologues of *Drosophila btd*. Mice deficient for either *Sp5 *or *Sp8 *have been reported. *Sp5 *knockout mice reportedly have no overt phenotypes [10]. In contrast, analyses of *Sp8 *knockout mice [9,11], complemented by retrovirus-mediated overexpression studies in embryonic chicks using dominant negative and active forms of *Sp8 *[8], indicate that *Sp8 *is required for limb and head formation.

A prominent role for *Sp8 *in limb development is to maintain the expression of several morphogens, including fibroblast growth factor (*Fgf*)*8*, bone morphogenic protein (*Bmp*)*4*, and Sonic hedgehog (*Shh*) [9,11]. Because these morphogens have prominent roles in forebrain development, we examined the expression of the *Sp8*-like family members in the developing mouse forebrain to determine their potential for roles in forebrain patterning, particularly the patterning of the neocortex into 'areas', which are anatomically and functionally distinct divisions with specialized functions in sensory perception, motor performance, learning and memory.

The genetic mechanisms that control cortical area patterning, that is, arealization, have come under intense study in the past few years, but remain sketchy. Current evidence indicates that morphogens secreted by signaling centers located at the perimeter of the developing dorsal telencephalon (dTel) establish gradients of TFs across the dTel ventricular zone (VZ), which in turn determine the area fate of cortical progenitors and their progeny [12-17]. These morphogens include Fgf8 expressed by the anterior neural ridge (ANR), which later becomes the commissural plate (CoP), located at the anterior midline of the dTel, and also members of the *wingless-int *(*Wnt*s) and *Bmp *families expressed by the cortical hem and choroid plexus epithelium (CPe), located at the dorsal midline of more posterior dTel. In addition, the morphogen Sonic hedgehog (*Shh*) is expressed in the forebrain, but in ventral structures, including the prechordal plate and the medial ganglionic eminence (MGE).

Here we describe that each of these three major signaling centers in mouse forebrain is uniquely associated with the discrete expression patterns of a member of the *Sp8*-like *btd *family, *Sp5*, *Sp8 *and *Sp9*. These associations are suggestive of functional interactions between the *Sp *family members and the respective morphogens. To address this issue, we focused on functions of *Sp8*, which at early stages of cortical development is expressed in association with the expression domain of *Fgf8 *in the ANR/CoP. We employed both gain and loss of function strategies using *in utero *electroporation to manipulate gene function in a localized manner, a technique similar to that recently used by others to show roles for *Fgf8 *in cortical area patterning [18,19]. We complemented these *in vivo *studies with *in vitro *assays of inductive pathways and interactions. These approaches allowed us to overcome the early embryonic lethality and forebrain defects exhibited by *Sp8 *knockout mice, including the failure of the anterior neural tube to close and the cerebral cortex to develop, that preclude an analysis of *Sp8 *in cortical patterning [9,11].

We show that *Sp8 *and *Fgf8 *can robustly induce one another, and that the induction of *Fgf8 *by *Sp8 *is repressed by the homeodomain TF, Emx2, which itself has been implicated in controlling area patterning [19-22]. The reciprocal induction between *Sp8 *and *Fgf8 *exhibits significant specificity among family members. In addition, we show that *Sp8 *affects cortical area patterning, but in a manner that opposes that of *Fgf8*, raising the possibility that Sp8 influences area patterning not only by regulating Fgf8 and its signaling pathway, but also through a distinct mechanism. Our findings show that *Sp8 *interacts with *Fgf8 *to balance the anterior-posterior (A-P) area patterning of the cortex.

## Results

### Forebrain expression of the *Sp8*-like family, mouse btd homologues, and correlation with signaling centers in the forebrain

**Figure 1:**
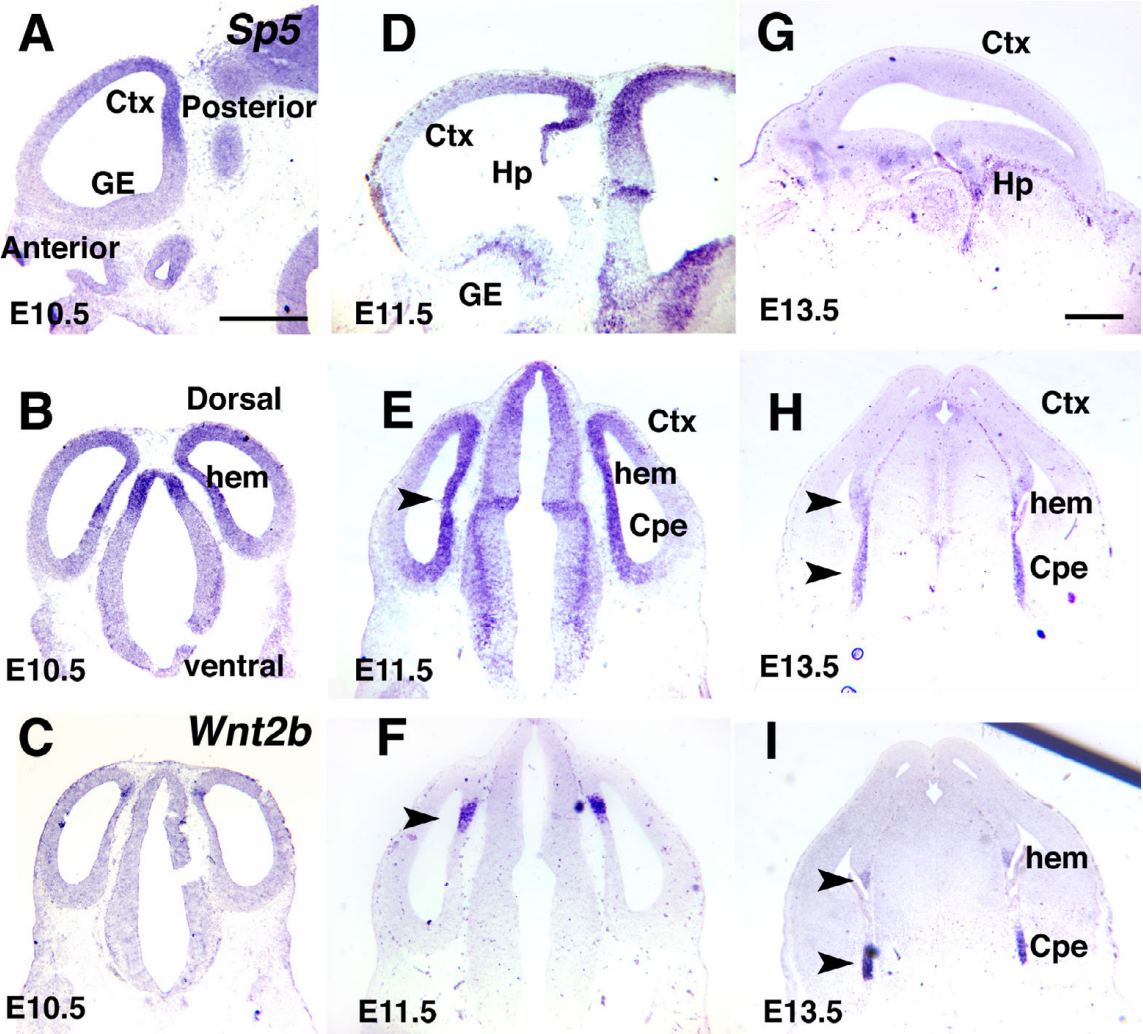
Expression patterns of Sp5 in embryonic mouse forebrain and relationship with cortical hem and choroid plexus. ** (a-i) **Sp5 expression relates to the cortical hem, a source of Wnts and Bmps. Shown are *in situ *hybridizations using DIG-labeled riboprobes for Sp5 on sagittal (a, d, g) or coronal (b, e, h) sections and Wnt2b (c, f, i) of mouse forebrain at E10.5 to E13.5. Sp5 expression is observed around the cortical hem and Cpe as being highest in medial and posterior parts of dorsal telencephalon (dTel; arrowhead), and quickly downregulated at E13.5. Unlike the expression of Sp8, Sp5 expression domains overlap those of Wnts throughout E10.5 to 13.5. Ctx, cortex; GE, ganglionic eminence; Hp, hippocampus. Scale bars: 0.5 mm

**Figure 2:**
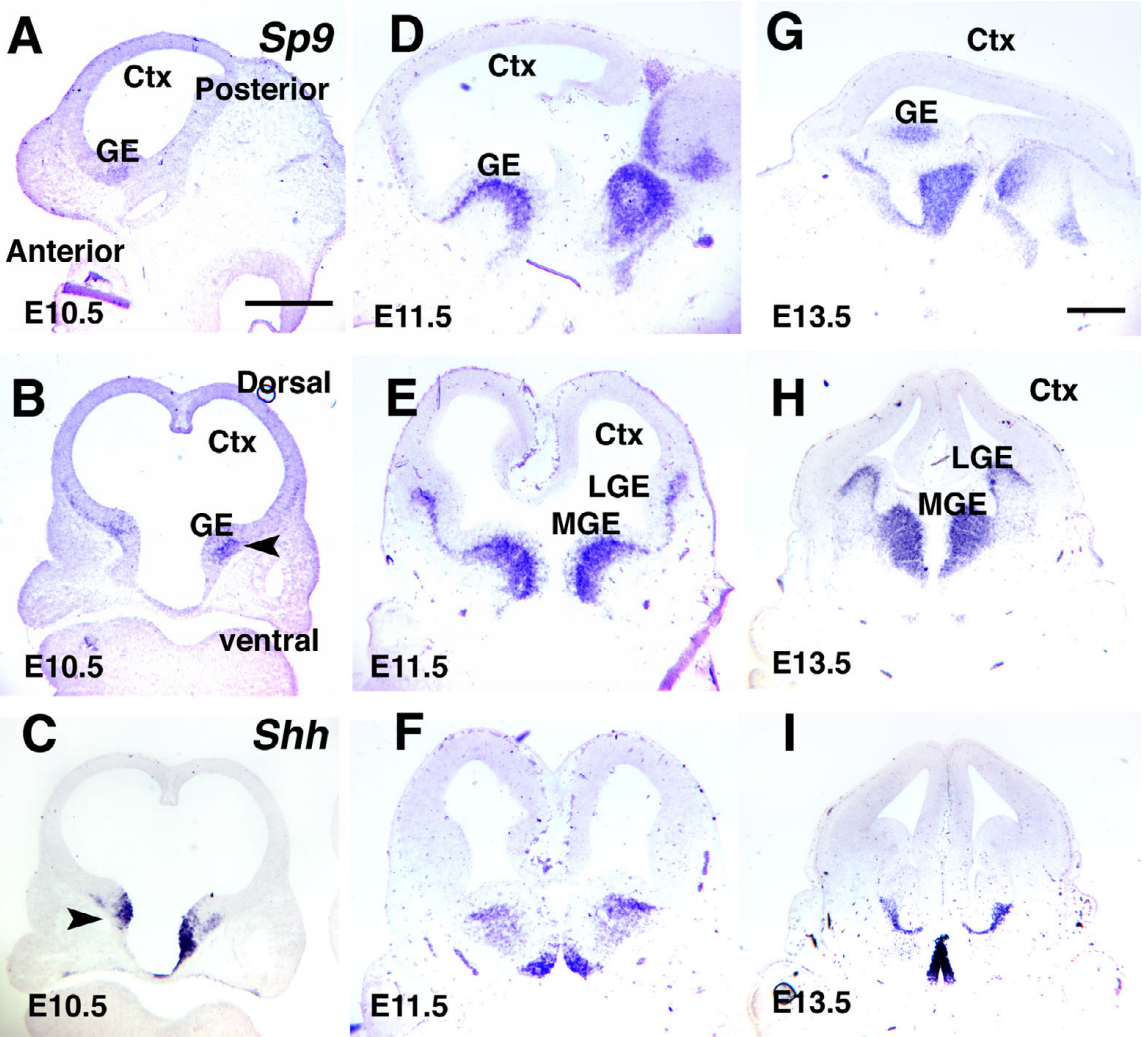
Expression patterns of Sp9 in embryonic mouse forebrain and relationship with the *Shh *expression domain in ventral telencephalon. **(a-i) **Comparison of Sp9 and Shh expression. Sp9 expression is not detected in cortex, but it is expressed highly in the SVZ of the MGE, the source of GABAergic interneurons that populate the cortex (arrowheads), and weakly in the LGE that populates interneurons migrating to olfactory bulbs. *Sp9 *expression domains are observed in the vicinity of the *Shh *expression domain, but does not overlap them. Ctx, cortex. Scale bars: 0.5 mm.

**Figure 3:**
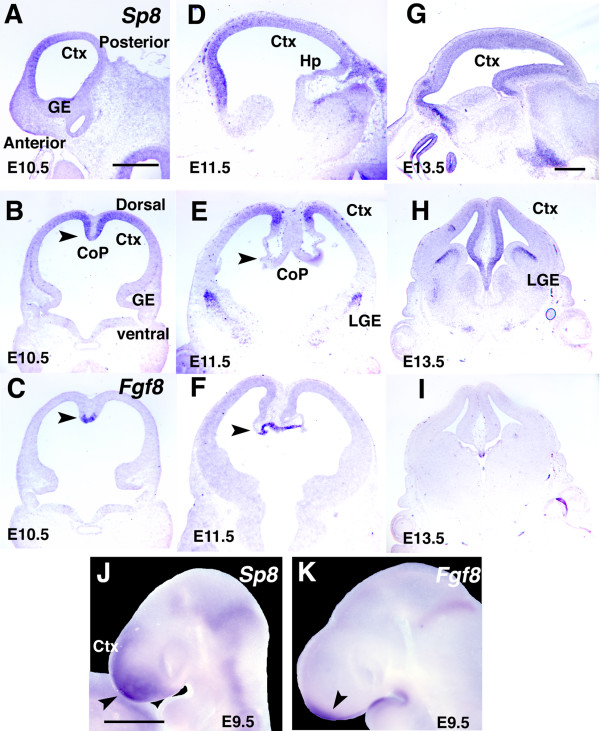
Expression patterns of Sp8 in embryonic mouse forebrain and relationships with expression domains of Fgf8 in the CoP. **(a-i) **Shown are *in situ *hybridizations of sections of mouse forebrain at E10.5 to E13.5 using DIG-labeled riboprobes for Sp8 on sagittal (a, d, g) or coronal (b, e, h) sections, and for Fgf8 (c, f, i). Sp8 is expressed in the dTel VZ, with highest levels in the medial and anterior parts. Sp8 is also expressed robustly in the LGE and at lower levels in the MGE. Sp8 expression overlaps the Fgf8 expression domain in the CoP at E10.5 (arrowhead in (b, c)), but gradually become excluded at E11.5 (arrowhead in (e, f)). **(j, k) **Whole mount *in situ *hybridizations done on E9.5 embryos with DIG-labeled riboprobes for Sp8 (j), and Fgf8 (k), respectively. The Sp8 A/P gradient is evident at this stage (j). Arrowheads indicate expression domains. Ctx, cortex; GE, ganglionic eminence; Hp, hippocampus. Scale bars: 0.5 mm (a-f); 0.5 mm (g-i); 0.5 mm (j, k).

We used *in situ *hybridization to analyze the expression patterns of the *Sp8*-like family members (*Sp5–Sp9*) on brain whole mounts at E9.5 and sections through the forebrain from E9.5 through E13.5 (Figure 1, 2, 3; data not shown). We did not detect expression of *Sp6 *and *Sp7 *(data not shown) [23,24]. At early stages of cortical development, E9.5 to E13.5, we detect expression of *Sp5 *and *Sp8 *in dTel within the cortical ventricular zone (VZ), whereas *Sp9 *expression is not detected within dTel but is strong in ventral telencephalon. As detailed in the following sections, each of these *Sp *expression domains correlates with a forebrain signaling center defined by the expression of unique sets of morphogens in the telencephalon.

### *Sp5 *expression

Between E9.5 and E11.5, expression of Sp5 is highest in the medial and posterior dTel, including the cortical hem and CPe, and exhibits a very steep gradient with the cortical VZ, quickly declining from high to low levels within more posterior and medial dTel, resulting in very low levels in more anterior and lateral dTel (Figure 1a, b, d, e, g, h). The pattern of Sp5 expression is considerably more extensive than the cortical hem/CPe, but most of the caudomedial part of the Sp5 expression domain contains the Wnt2b expression domain (Figure 1c, f, i), a marker for the cortical hem [16]. By E13.5, Sp5 expression declines to non-detectable levels in the cortex, but persists in the hem and CPe (Figure 1). Sp5 is also expressed at lower levels in the mantle of the MGE.

### *Sp9 *expression

At E10.5, *Sp9 *is modestly expressed in the mantle zone of the ganglionic eminence (GE) (Figure 2a, b) and, therefore, likely in postmitotic neurons. At E11.5 (Figure 2d, e), *Sp9 *is robustly expressed in the MGE, throughout the mantle zone and likely also in the subventricular zone (SVZ) of the MGE. At this age, the MGE mantle includes postmitotic GABAergic interneurons that migrate into and populate the cortex [25], and the SVZ contains proliferating cells. *Sp9 *is also expressed robustly in the SVZ of the lateral GE (LGE). *Sp9 *expression overlaps with *Shh *expression in the MGE mantle. However, elsewhere in ventral telencephalon, *Shh *is predominantly expressed in largely non-overlapping, complementary patterns with *Sp9 *(Figure 2).

### *Sp8 *expression

From E9.5 through E13.5, *Sp8 *is expressed in a high to low medial to lateral gradient across the dTel VZ, and is expressed across the A-P axis of dTel, with a shallow high to low A-P gradient; by E10.5, *Sp8 *is expressed across the entire A-P axis. The *Sp8 *domain is associated with the *Fgf8 *expression domain in the CoP (Figure 3b, c). The correlation of the *Sp8 *domain with the *Fgf8 *expression domain in the CoP is dynamic over the brief time window of *Fgf8 *expression (Figure 3b, c, e, f, h, i). At E9.5 and E10.5, the *Sp8 *and *Fgf8 *domains overlap, although the *Sp8 *domain is more expansive and extends laterally and posteriorly from the CoP through the dTel VZ (Figure 3b, c, j, k). At E11.5, *Sp8 *is robustly expressed, but it is beginning to segregate from the *Fgf8 *domain at the posterior domain, with *Fgf8 *largely limited to the midline CoP, and *Sp8 *largely excluded from it and expressed in a high to low gradient extending laterally from the midline (Figure 3e, f). At E13.5, *Sp8 *expression in the dTel VZ is still present but substantially diminished, and *Sp8 *has become excluded from the increasingly restricted *Fgf8 *domain in the CoP (Figure 3h, i).

*Sp8 *is also expressed in the SVZ of the LGE, a source of interneurons that migrate into the olfactory bulbs, and at low levels in the mantle of the LGE and MGE. *Sp8 *has been shown to regulate the specification, survival and migration of olfactory interneurons [26].

### *Sp8 *and *Fgf8 *exhibit reciprocal induction

**Figure 4:**
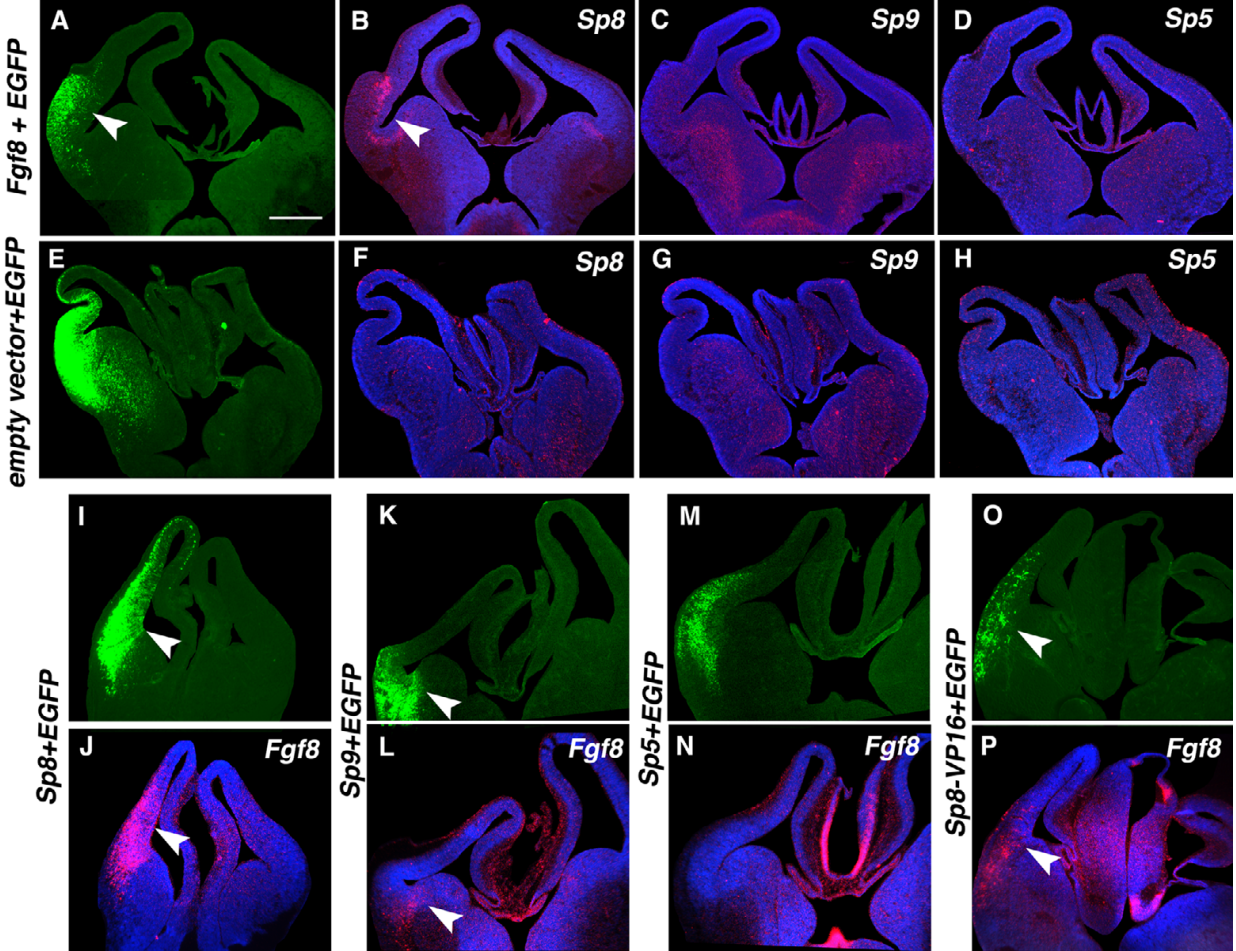
Reciprocal induction between Sp8 and Fgf8 *in vivo*. **(a-h) **Induction of Sp8 by ectopic expression of Fgf8. Coronal sections of E13.5 forebrains that were electroporated *in utero *at E11.5 with Fgf8b (a-d) or a control vector (e-h) mixed with EGFP and processed for *in situ *hybridization using S^35 ^riboprobes for Sp5 (d, h), Sp8 (b, f) and Sp9 (c, g), respectively. Induction of Sp8 was detected in the electroporated domain marked by EGFP (a, e) (arrowheads). Sp5 and Sp9 were not induced by ectopic expression of Fgf8 (c, d). **(i-p) **Induction of Fgf8 by ectopic expression of Sp8 or the dominant active form of Sp8 (Sp8-VP16). E13.5 brains were electroporated with Sp8 (i, j), Sp9 (k, l), Sp5 (m, n), or Sp8-VP16 (o, p), mixed with EGFP, and processed for *in situ *hybridization using Fgf8 riboprobes. Fgf8 induction was detected in Sp8 (j) and Sp8-VP16 (p) electroporated brains. Weak induction of Fgf8 was also observed by the electroporation of Sp9 (l), which shares identical btd and Zn-finger domains with Sp8. Fgf8 induction was not detected in brains electroporated with Sp5 (n). Scale bars: 0.5 mm (a-p).

For functional analyses, we have focused on *Sp8 *and its relationship with the *Fgf8 *expression domain in the CoP, the potential for an interaction between Sp8 and Fgf8, and its influence on cortical area patterning. The expression pattern of *Sp8 *suggests that *Sp8 *might be a target of *Fgf8*, which is consistent with the finding that *Sp8 *expression is diminished in conditional or hypomorphic *Fgf8 *knockout mice [27]. To address whether Fgf8 induces *Sp8*, we ectopically expressed *Fgf8 in vivo *in the telencephalon by *in utero *electroporation of a CAG *-Fgf8 *expression vector; in this, and all other similar experiments, a enhanced green fluorescent protein (EGFP) expression vector was co-electroporated as a reporter to mark the transfection domain. Electroporations were done on E11.5 and the brains analyzed at E13.5. Adjacent sections were processed by *in situ *hybridization for ectopic expression of *Sp8*, *Sp9 *and *Sp5*. *Sp8 *is consistently induced by ectopic *Fgf8*, (n = 8 of 9 cases, Figure 4b), whereas we do not detect induction of ectopic expression of either *Sp9 *(n = 4 of 4, Figure 4c) or *Sp5 *(n = 4 of 4, Figure 4d). The control vector has no influence on *Sp *expression (n = 4 of 4, Fig 4f–h. These findings show that *Fgf8 *induces *Sp8*, indicating that *Sp8 *is a downstream target of *Fgf8 *but *Sp5 *and *Sp9 *are not.

To address whether *Sp8 *reciprocally induces *Fgf8*, we ectopically transfected expression vectors for *Sp8*, or to assess specificity, *Sp5 *and *Sp9*. We found that ectopic expression of *Sp8 *robustly induces *Fgf8 *expression (n = 9 of 10, Figure 4j). Ectopic expression of *Sp9 *only modestly induces *Fgf8 *(n = 8 of 9, Figure 4l), whereas *Sp5 *does not induce detectable levels of *Fgf8 *(n = 2 of 2, Figure 4n). These findings show that *Sp8 *can induce *Fgf8*, and, therefore, that *Sp8 *and *Fgf8 *form a reciprocal inductive loop.

To address whether *Sp8 *is a transcriptional activator of *Fgf8*, we used a strategy of fusing the *btd *and Zn finger functional binding domains of Sp8 to a *VP16 *domain, which is a potent transcriptional activator [8]. This chimeric construct acts as a dominant active form of *Sp8 *that can induce *Fgf8 *in the developing chick limb [8]. We found that ectopic expression of the *Sp8-VP16 *construct also induces *Fgf8 *(n = 12 of 14, Figure 4p ), indicating that *Sp8 *is a transcriptional activator of *Fgf8*. The *VP16 *construct alone (n = 6 of 6, data not shown) does not induce *Fgf8*. In summary, we demonstrate that *Fgf8 *and *Sp8 *exhibit reciprocal induction *in vivo *in the embryonic telencephalon. Furthermore, our findings show that *Sp8 *regulates transcription of *Fgf8*.

*Fgf17 *is expressed within the CoP, albeit in a broader domain than Fgf8, and has a similar effect as *Fgf8 *on A-P cortical patterning [18]. Therefore, we also performed *in utero *electroporations to test whether *Sp8 *and *Fgf17 *exhibit reciprocal induction. However, we did not detect reciprocal induction (n = 3 of 3 cases, data not shown), suggesting that *Sp8 *is not directly relevant to *Fgf17 *signaling. Thus, *Sp8 *and *Fgf17 *do not induce one another.

In conclusion, the reciprocal induction between *Sp8 *and *Fgf8 *exhibits a specificity that does not extend significantly to related *Fgf *and *Sp8*-like family members.

### *Sp8 *activates the *Fgf8 *signaling pathway

**Figure 5:**
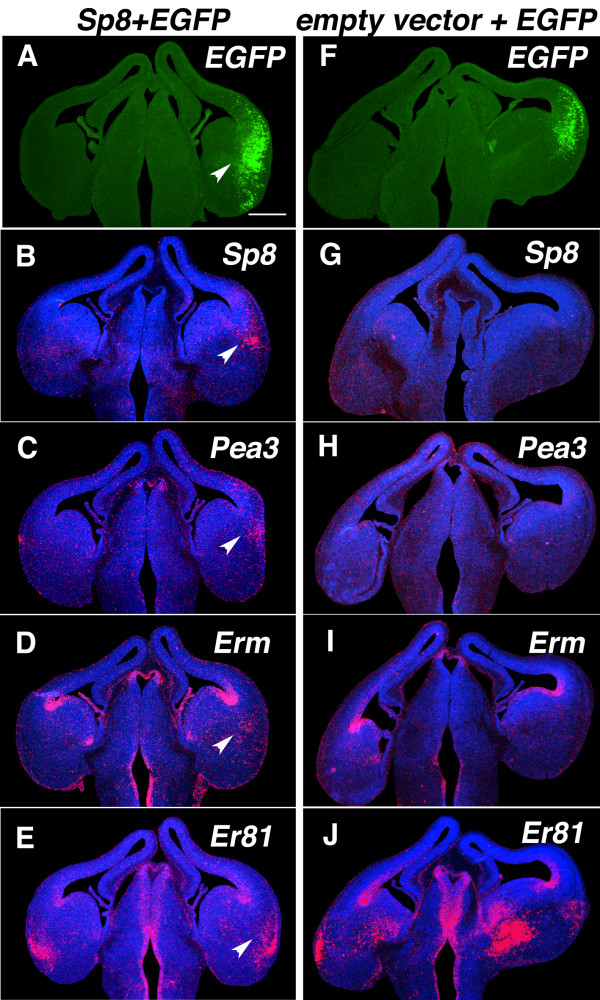
Sp8 induces Fgf8 downstream targets. (a-j) Induction of ectopic ETS TFs, Pea3, Erm and Er81 by the ectopic expression of Sp8. Coronal sections of E13.5 forebrains that were electroporated at E11.5 with Sp8 (a-e) or a control vector (f-j), mixed with EGFP, and processed for *in situ *hybridization using S^35 ^riboprobes for Sp8 (b, g), Pea3 (c, h), Erm (d, i) and Er81 (e, j), respectively. EGFP (a, f) marks the electroporation domains (arrowheads). Pea3 (c), Er81 (d) and Erm (e) were induced by ectopic expression of Sp8. Scale bars: 0.5 mm.

Our findings show that *Sp8 *is a transcriptional activator of *Fgf8*, suggesting that ectopic *Sp8 *induction of *Fgf8 *should also induce the *Fgf8 *downstream targets, including Fgf8-target genes such as the ETS (E-twenty-six) family TFs, *Pea3*, *Erm*, and *Er81 *[19]. To address this issue, we carried out additional *in utero *electroporations of *Sp8 *expression vectors as described above on adjacent sections through the transfection site identified by the GFP signal, and performed *in situ *hybridization for *Fgf8 *as well as ETS family members (Figure 5). Ectopic expression of *Sp8 *does indeed induce the expression of *Pea3 *(n = 4 of 4, Figure 5c), *Erm *(n = 3 of 3, Figure 5d), and *Er81 *(n = 3 of 3, Figure 5e), but coincident to the *Sp8-*transfection domains. Control vectors had no effect (n = 3 of 3, Figure 5g–j). These findings indicate that *Sp8 *not only induces *Fgf8*, but also components of its signaling cascade, suggestive of the activation of a functional *Fgf8 *signaling pathway.

### *Sp8 *binds the *Fgf8 *promoter and activates *Fgf8 *expression

**Figure 6:**
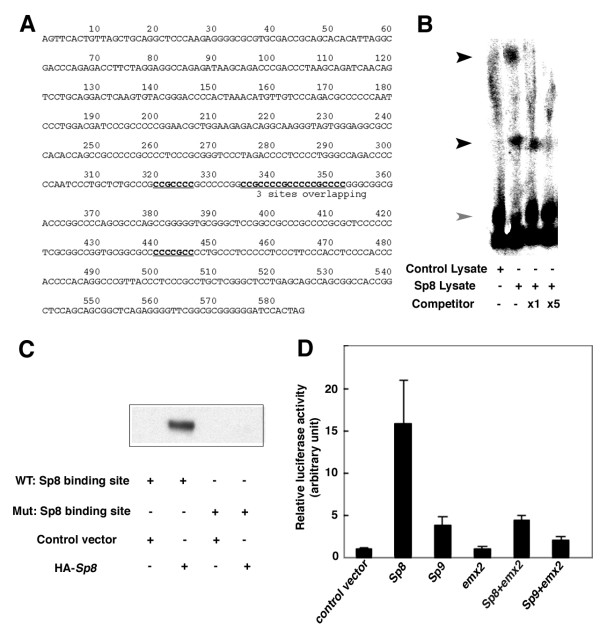
Sp8 directly binds the *Fgf8 *promoter and Emx2 represses Sp8 induction of Fgf8. (a) Sequence of a 585 bp fragment, containing the 555 bp immediate upstream region of the mouse *Fgf8 *transcription start site and the 30 bp 5' UTR of Fgf8 having six putative Sp8 binding sites. Putative Sp8 binding sites predicted from Sp1-binding motifs (GGGGCGG or CCCCGCC) are underlined. (b) Gel retardation assay of *Sp8 *and *Fgf8 *promoter fragments. P^32^-labeled *Fgf8 *promoter fragments show slow mobility after incubation with the Sp8-expressed lysate (black arrow) compared to the unbound DNA (gray arrow). These bands are not detected in the control lysate sample and are diminished in the presence of an equal (×1) or five-fold (×5) amount of unlabeled DNA compared to labeled probes. (c) Sp8 binding to the oligonucleotide of the *Fgf8 *promoter region. HA-Sp8 was co-precipitated with biotinylated non-mutated oligonucleotide corresponding to a putative Sp8 binding site, but not with oligonucleotides with a mutated core recognition sequence. (d) Luciferase reporter assay for induction of *Fgf8*. Control or test vectors (Sp8, Sp9, Emx2) were transfected independently or in combinations into C3H10T1/2 cells also transfected with an *Fgf8 *reporter construct consisting of a promoter element for *Fgf8 *and a firefly luciferase reporter vector. The relative effectiveness of Fgf8 induction was assessed by measuring luciferase activity. Sp8 robustly induces expression of the *Fgf8 *reporter construct; this Sp8 induction is suppressed by co-expression of Emx2. Sp9 modestly induces expression of the *Fgf8 *reporter construct; again, this is suppressed by Emx2. Emx2 alone has no effect on *Fgf8 *induction, similar to a control empty vector.

To gain more insight into the regulation of *Fgf8 *expression, we searched the genomic sequence of *Fgf8 *for putative Sp8 regulatory elements. Because Sp8 is a member of the Sp1-Zn finger family, we expected that Sp8 would bind DNA sequences similar to those described for Sp1 [28]. Within the 585 base-pairs (bp) upstream of the *Fgf8 *transcription start site, we identified six putative Sp8 binding sites, and a position at which three of six putative sites overlap with one another (Figure 6a).

To show Sp8 binding to *Fgf8 *promoter sequences, we first carried out a gel retardation assay. This 585 bp fragment of the *Fgf8 *promoter region was labeled with P^32 ^and incubated with lysate from reticulocytes expressing the Sp8 protein under the control of a T7 promoter, or control lysate. We detected two different bands in the Sp8 lysate but not in the control lysate (Figure 6b). To corroborate the specificity of these results, we performed a competition experiment by repeating the gel retardation assay as above, but in the presence of an excess of the 585 bp DNA fragment not labeled with P^32^. In this competition assay, the bands were absent, indicating that Sp8 protein directly binds to these fragments of the *Fgf8 *promoter region.

As a complementary approach, we tested Sp8 binding to the *Fgf8 *promoter sequences by a pull-down assay. Lysates from cells transfected with hemagglutinin (HA)-tagged Sp8 or control vector were incubated with a biotinylated oligonucleotide corresponding to the putative Sp8 binding site, or with one mutated at the core region of the Sp1-recognition sequences. We found co-precipitation of Sp8 with the wild-type oligonucleotide but not with the mutated version (Figure 6c). Thus, these data indicate that Sp8 recognizes and binds to this region of the *Fgf8 *promoter defined by sequence similarity to Sp1 binding sites.

To further investigate if Sp8 controls *Fgf8 *expression through binding this promoter region, we used a luciferase reporter assay (Figure 6d). Control or test vectors containing *Sp8*, *Sp9*, or *Emx2 *were transfected independently or in combinations into C3H10T1/2 cells also transfected with an *Fgf8 *reporter construct consisting of the same 585 bp promoter element for *Fgf8 *used in the gel retardation assay, and a firefly luciferase reporter vector [29]. Transfection of an *Sp8 *expression vector robustly induces expression of the *Fgf8 *reporter construct, whereas *Sp9 *has only a modest inductive effect.

Noggin, a Bmp inhibitor, has been recently reported to induce *Fgf8 *expression when overexpressed in forebrain [30]. Therefore, we tested whether a suppression of Bmp signaling could contribute to Sp8-mediated Fgf8 upregulation. However, we found that manipulation of Bmp activity has no effect on Sp8 transcriptional activation of Fgf8 in the *in vitro *reporter assay (Additional file 1).

Taken together these findings show that Sp8 binds to a proximal promoter region of the *Fgf8 *gene of *Fgf8*, and that this binding induces *Fgf8 *expression.

### *Sp8 *induction of *Fgf8 *is repressed by Emx2

As described above, *Sp8 *is expressed in the CoP coincident with *Fgf8*. However, *Sp8 *is also expressed outside of the CoP, within the VZ of dTel (neocortex), locations where *Fgf8 *is not expressed, suggesting that factors expressed in the cortical VZ repress the ability of *Sp8 *to induce *Fgf8 *and restrict its expression to the CoP. *Emx2 *is a candidate repressor because the domain of *Fgf8 *expression in the CoP is broader in *Emx2 *null mice, and ectopic *Emx2 *expression in the CoP in slice culture suppresses *Fgf8 *expression [19].

Therefore, we tested whether Emx2 can repress *Sp8 *induction of *Fgf8 *in a luciferase reporter assay. An *Emx2 *construct transfected alone has no effect on expression of the Fgf8 reporter, and is indistinguishable from a control empty vector alone. However, when *Emx2 *is co-transfected with *Sp8*, *Emx2 *represses *Sp8 *induction of the *Fgf8 *reporter construct. *Emx2 *also represses the modest induction of *Fgf8 *exhibited by *Sp9*.

In summary, these findings are consistent with our *in vivo *analyses, and provide a molecular mechanism for them, demonstrating that *Sp8 *binds *Fgf8 *regulatory elements and is likely a direct transcriptional activator of *Fgf8*. Furthermore, they show that Emx2 can act as a repressor of Sp8 induction of *Fgf8*.

### *Sp8 *is required to maintain *Fgf8 *expression *in vivo*

**Figure 7:**
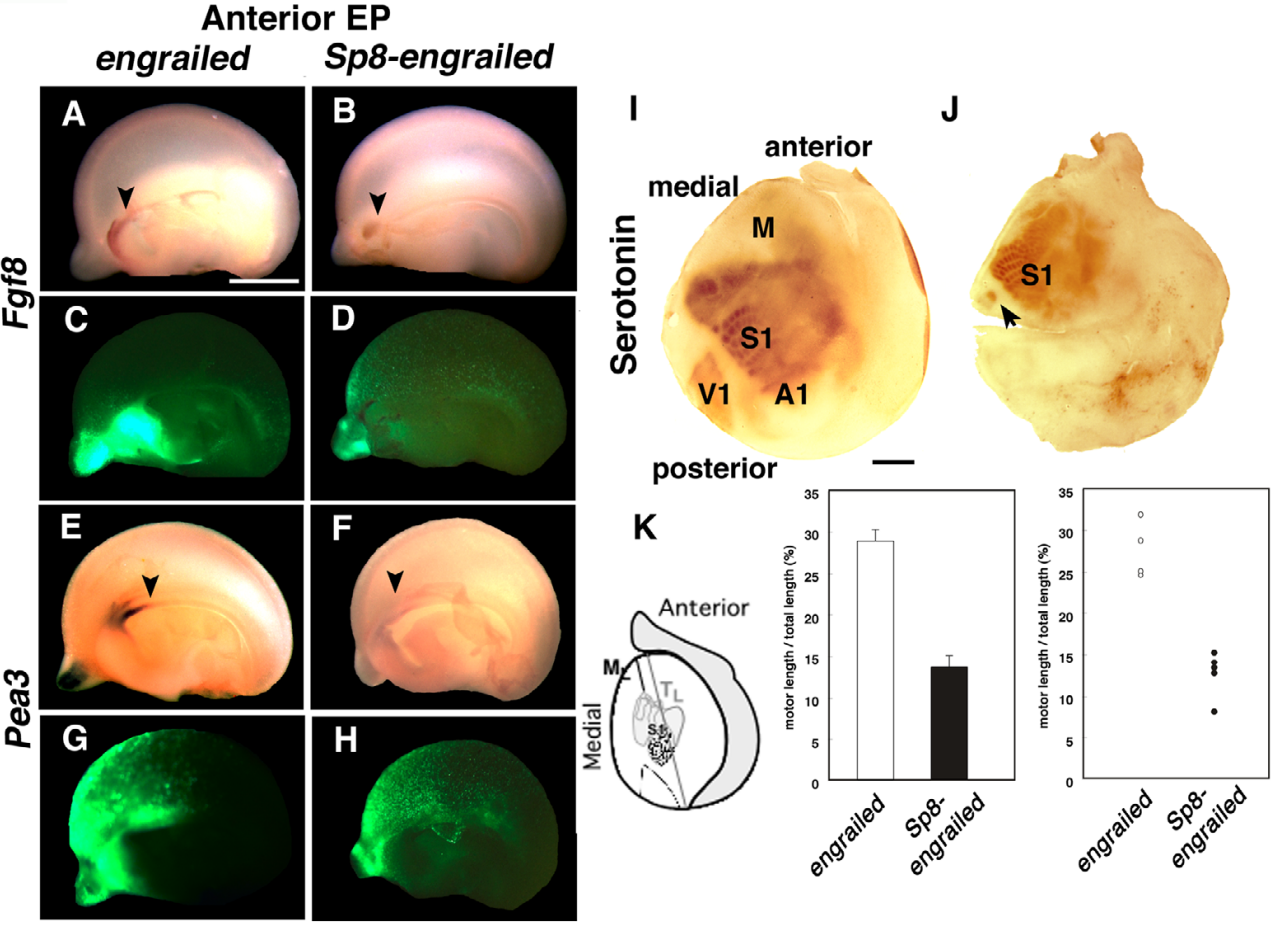
Anterior electroporation of a dominant negative form of *Sp8 *inhibits endogenous Fgf8 expression in the commissural plate and results in an anterior shift in area patterning. Anterior electroporation of a dominant negative form of *Sp8 *inhibits endogenous Fgf8 expression in the commissural plate and results in an anterior shift in area patterning. **(a-h) **Anterior electroporation of a dominant negative form of *Sp8 *inhibits endogenous Fgf8 expression in the CoP. Electroporation of the *Sp8 *dominant negative chimeric construct, *Sp8-engrailed*, suppresses endogenous Fgf8 expression in the CoP (b), as well as expression of Pea3, a downstream target of Fgf8 (f) at E14.5. Fgf8 and Pea3 expression in brains with similar electroporation of the control engrailed construct (a, e) is indistinguishable from non-transfected brains (not shown). Arrowheads indicate Fgf8 (a, b) and Pea3 expression domains (e, f). An EGFP expression construct co-electroporated with *Sp8-engrailed *and *engrailed *control constructs are shown in parallel and define the transfection domain (c, d, g, h). **(i, j) **Anterior ectopic expression of *Sp8-engrailed *results in an anterior shift of cortical areas. Tangential cortical sections through layer 4 of a P7 brain electroporated at E11.5 with control vector (i) or Sp8-engrailed (j) and processed for serotonin immunostaining to reveal the primary sensory areas: somatosensory (S1), auditory (A1) and visual (V1). In cortex of brains with anterior electroporation of *Sp8-engrailed*, S1 shifts far anteriorly, resulting in a very small amount of cortex remaining for motor (M) areas. The arrow in (j) indicates putative visual areas. Scale bars: 0.5 mm (a-h), 1.0 mm (i, j). **(k) **Quantification of motor area size in *Sp8-engrailed *electroporated brains. The length of motor areas is measured as a ratio of the length from the edge of anterior flattened brains to the staining of S1 regions (M_L_) and the total length (T_L_) of serotonin staining of flattened brains. Compared to *engrailed *control vector cases (28.89 ± 1.35% standard error of the mean (SEM, n = 5), brains electroporated with *Sp8-engrailed *show shrunken motor areas (13.66 ± 1.34% SEM, n = 6), as indicated in the middle panel. The right panel shows a scatter plot of individual cases.

To obtain further evidence that *Sp8 *regulates *Fgf8 *expression *in vivo*, we used a dominant negative strategy by fusing the *btd *and Zn finger DNA binding domains of Sp8 to an engrailed repressor domain, which has been shown in developing chick limb to act as a dominant negative form of *Sp8 *[8]. We electroporated the *Sp8*-engrailed construct in the anterior domain of dTel at E11.5, and analyzed expression at E14.5. An engrailed control vector, consisting of the engrailed repressor sequences but lacking the *Sp8 *DNA binding domains has no effect on *Fgf8 *expression when transfected into the CoP (n = 9 of 9, Figure 7a). In dramatic contrast, transfection of the dominant negative *Sp8-engrailed *construct into the CoP markedly suppresses *Fgf8 *expression in this anterior domain, although intense GFP expression is evident (n = 10 of 13, Figure 7b).

These results were also confirmed by the expression of *Pea3*, a member of the *Pea3 *ETS TF subfamily that is rigidly regulated by *Fgf8 *[31,32]. We found that transfection of the dominant negative *Sp8-engrailed *construct into the CoP results in a substantially diminished expression of *Pea3 *(n = 7 of 8, Figure 7f), whereas the control engrailed construct has no effect (n = 9 of 9, Figure 7e). These results demonstrate that *Sp8 *is required to maintain *Fgf8 *expression within the CoP *in vivo*, as well as expression of its targets.

### Maintained expression of *Fgf8 *by *Sp8 *is required for proper cortical area patterning

If *Sp8 *is required to maintain *Fgf8 *expression and activation of its signaling pathway, as our results above show, then electroporation of the dominant negative form of *Sp8 (Sp8*-*engrailed*) into anterior dTel should affect cortical area patterning by diminishing *Fgf8 *signaling. We predict that the effect on area patterning should resemble the anterior area shifts produced by ectopic expression in anterior dTel of the extracellular domain of *Fgf *receptor 3c (*sFGFR3c*), which is interpreted to be due to *sFGFR3c *binding and suppressing the action of endogenous *Fgf8 *protein secreted by the CoP [18].

To test this prediction, we performed *in utero *electroporations of the *Sp8-engrailed *construct into anterior dTel at E11.5 as above, but rather than analyzing the brains at embryonic ages, we analyzed their area patterning at P7 using serotonin immunostaining on tangential sections, which clearly delineate the primary sensory areas [33]. We observed that *Sp8-engrailed *electroporation induces a substantial shift of area patterns toward the anterior pole of the cortex and a substantial reduction in the sizes of the primary sensory areas (n = 6 of 6, Figure 7j). Electroporation of the control engrailed domain alone does not affect area patterning (n = 5 of 5, Figure 7i). Thus, these anterior area shifts are consistent with an *Sp8-engrailed *mediated repression of targets of *Sp8*, such as *Fgf8 *and its downstream effectors (for example, *Pea3*). Thus, *Sp8 *is crucial for maintaining *Fgf8 *expression in the CoP and activation of its signaling pathway, as well as the influence of Fgf8 signaling on cortical arealization.

### Changes in area patterning induced by ectopic expression of *Fgf8 *

As a prelude to addressing the effect of ectopic expression of *Sp8 *on area patterning, we performed *in utero *electroporation of a *CAG-Fgf8 *expression construct into either anterior or posterior dTel at E11.5, and analyzed area patterning at P7 using serotonin immunostaining on tangential sections [33]. These cases allowed us to compare the effect of ectopic expression of *Sp8 *to that of *Fgf8 *done in the same manner.

In brains with electroporations done at E11.5 and examined at E13.5/E14.5, about 80% of the transfection domains marked by the EGFP reporter are located at the region of dTel targeted for electroporation (anterior pole or posterior pole) (105 of 137 cases). However, because the EGFP reporter co-electroporated at E11.5 with the *Sp8 *constructs is no longer detectable at P7, to confirm these results, we repeated these studies using ROSA26 reporter mice, and co-electroporated the *Sp8-VP16 *dominant active construct or the *VP16 *control construct with a CAG-Cre Recombinase construct; the CAG-Cre Recombinase activates the β-gal reporter to mark the lineage of transfected cells and hence the location of the transfection domain. The findings confirm that the transfection domains, marked by β-gal labeled cells, are at positioned where the transfection was targeted, either the anterior or posterior cortical pole (Additional file 2), and the area shifts are identical to those described above

**Figure 8:**
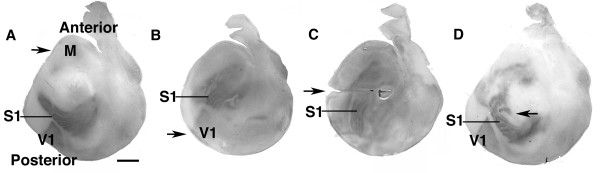
Cortical area shifts by ectopic expression of *Fgf8 *at anterior or posterior sites. Electroporation of a *CAG-Fgf8 *vector was done at E11.5 and later, at P7, the brains were processed and tangential sections of their cortical areas were visualized by serotonin staining as described in Methods. (a) Ectopic expression of *Fgf8 *at an anterior site in E11.5 brains causes posterior area shifts with motor (M) area expansion (n = 2 of 2 electroporated cases; this finding replicates that of Fukuchi-Shigomori and Grove [18], therefore we did not perform additional cases) compared to control electroporated brains. (b) In contrast, a posterior electroporation of *Fgf8 *induces anterior area shifts accompanied by an expansion of V1 (n = 4 of 6 electroporated cases). We also performed electroporations using medial and lateral approaches at mid-posterior levels in an attempt to target the presumptive barrelfield of S1 (posteromedial barrel subfield, PMBSF). (c, d) Medial-posterior electroporation of *Fgf8 *typically causes an 'elongated' S1 (c) (n = 7 of 10 electroporated cases), whereas lateral-posterior electroporation within the cortical field that would develop as S1 can result in a 'split barrel field' (d) (n = 2 of 9 electroporated cases). The middle of the 'PMBSF' in S1 is pointed to by the line. We did not observe 'duplicate' barrels as reported by Fukuchi-Shigomori and Grove [18]. We assume that duplicated barrels are produced only in a unique situation with an appropriate combination of timing, size and position of the ectopic *Fgf8 *expression domain, and level of Fgf8 expression, required to partially duplicate the barrel pattern. Arrows indicate targeted locations of the electroporation sites. M, motor areas. Scale bar: 1.0 mm.

Anterior electroporation of *Fgf8 *causes a substantial expansion of the anterior cortical field, which includes motor areas (Figure 8a), as previously reported [18]. In contrast, we observed that posterior electroporation of *Fgf8 *induces an anterior shift of areas accompanied by an expansion of V1 (Figure 8b). Electroporations of *Fgf8 *that targeted the S1 barrelfield, made from a medial or lateral approach at a mid-posterior level, resulted in aberrant patterning of the barrelfield and shifts in its position (Figure 8c, d). Thus, ectopic expression of Fgf8 in anterior dTel results in a posterior shift in cortical areas, whereas ectopic expression of Fgf8 in posterior dTel results in an anterior shift.

### *Sp8 *induces area shifts that oppose those induced by *Fgf8 *

Because both full-length and dominant active *VP-16 *forms of *Sp8 *induce *Fgf8 *and its signaling pathway, we expected that ectopic expression of *Sp8 *should have an effect on cortical arealization resembling that produced by ectopic expression of *Fgf8*. Therefore, we predicted that anterior electroporation of these *Sp8 *constructs would result in a posterior shift of cortical areas, similar to that for anterior overexpression of *Fgf8 *[18] (Figure 8a). Based on similar logic, we anticipated that posterior ectopic expression of the *Sp8 *constructs should result in an anterior shift of cortical areas. We performed *in utero *electroporations of the *Sp8 *constructs into anterior or posterior dTel at E11.5, and analyzed area patterning at P7 using serotonin immunostaining on tangential sections [33].

**Figure 9:**
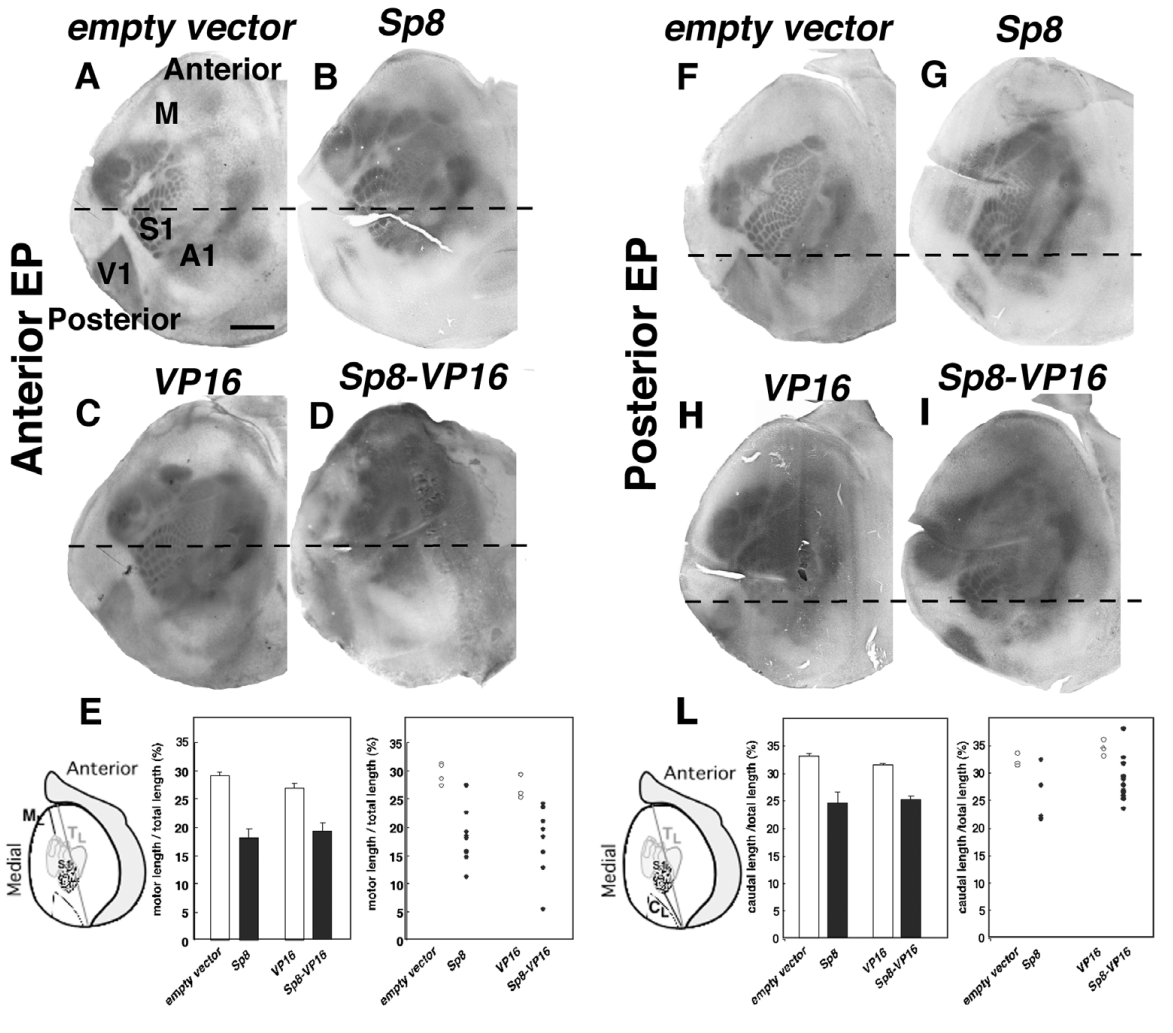
Ectopic expression of Sp8 and a dominant active form of Sp8 induce area shifts that oppose Fgf8. **(a, b) **Ectopic expression of Sp8 (b) at an anterior site of E11.5 brains causes anterior area shifts with a substantial reduction of motor (M) areas compared to control electroporated brains (a) that are indistinguishable from wild type (not shown). Areas of tangential section at P7 cortices are visualized by serotonin immunostaining. **(c, d) **Anterior ectopic expression of a dominant active form of *Sp8*, *Sp8-VP16 *(d), but not theVP16 domain alone (c), induces anterior area shifts similar to Sp8. The dashed line is at the E1 barrel position of control brain for the A-P comparison. **(e) **Quantification of motor areas in *Sp8*-, *Sp8-VP16*, or control-transfected brains. Anterior electroporation of either *Sp8 *or *Sp8-VP16 *causes reduction of the motor areas ratio (Sp8, 18.07 ± 1.52% SEM, n = 10; Sp8-VP16, 19.12 ± 2.18% SEM, n = 9), compared to the control cases (control vector; 29.05 ± 0.691%, SEM, n = 5; VP16, 26.88 ± 0.853%, SEM, n = 5). The right panel shows a scatter plot of individual cases. **(f-i) **Posterior ectopic expression of *Sp8 *and *Sp8-VP16 *induces posterior cortical area shifts. Sp8 (g) and Sp8-VP16 (i) ectopic expression at posterior sites causes a posterior shift in area patterning with a reduction in size of V1 areas, whereas expression of an empty vector (F) or the VP16 domain alone (H) has no effect on area patterning. The dashed line is at the A1 barrel position of control brain. Scale bar: 1.0 mm. **(l) **Quantification of caudal areas in *Sp8*-, *Sp8-VP16*, or control-transfected brains. Posterior electroporation of either *Sp8 *or *Sp8-VP16 *causes reduction of caudal area ratio (Sp8, 24.42 ± 2.03%, SEM, n = 6; Sp8-VP16, 24.89 ± 0.700% SEM, n = 18), compared to the control cases (control vector, 33.02 ± 0.581%, SEM, n = 5; VP16, 31.16 ± 0.430%, SEM, n = 5). The right panel shows a scatter plot of individual cases. EP, electroporation; M_L_, motor length; C_L_, caudal length;T_L_, total length.

Contrary to our prediction, we found that anterior electroporation of *Sp8 *results in an anterior shift of areas (n = 8 of 10, Figure 9b) judging from serotonin staining of tangential sections at P7, while similar electroporation of the control construct (n = 5 of 5, Figure 9a) results in an area pattern that is indistinguishable from wild type. Anterior electroporation of the *Sp8*-*VP16 *also results in an anterior shift of areas (n = 7 of 9, Figure 9d), whereas anterior electroporation of the *VP16 *control construct (n = 5 of 5, Figure 9c) again results in an area pattern that is indistinguishable from wild type. Because these area shifts oppose those produced by anterior overexpression of *Fgf8*, they suggest that although *Sp8 *can induce *Fgf8*, *Sp8 *might also activate distinct pathways to overcome *Fgf8 *signaling for area patterning

Posterior electroporations of either the full-length *Sp8 *or *Sp8-VP16 *constructs result in a posterior shift of areas, accompanied by a substantial reduction in the size of V1, the most posterior primary sensory area (*Sp8*, n = 5 of 6; *Sp8*-*VP16*, n = 16 of 18; Figure 9h, l); again, control electroporations have no effect on area patterning. This finding is consistent with the anterior shift produced by anterior electroporation of *Sp8*. However, these area shifts induced by *Sp8 *(Figure 9) are the opposite of those observed when we electroporate an *Fgf8 *expression construct in posterior dTel, which we find produces an anterior shift in cortical areas (Figure 8).

### Area shifts by *Sp8 *are paralleled by changes in markers of positional identity

To address whether area changes induced by *Sp8 *are accompanied by changes of positional identity of cortical neurons, we carried out a marker analysis using *cadherin8 *(*cad8*), *RORβ*, and *ephrin-A5*, each of which exhibit differential expression patterns that develop independent from thalamocortical axon input, indicating that they are *bona fide *markers of an intrinsic positional identity of cortical neurons [34,35]. *Cad8 *expression was detected at P1 by whole mount *in situ *hybridization using digoxygenin-labeled probes, and *RORβ *and ephrin-A5 were detected using S^35 ^labeled probes on sagittal sections. Expression was analyzed in P1 brains following *in utero *electroporation with *Sp8 *expression vectors or control vectors in anterior dTel or posterior dTel at E11.5.

**Figure 10:**
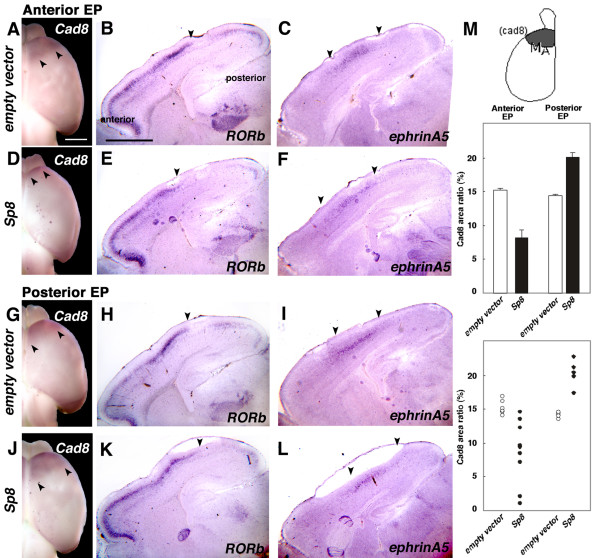
Sp8 changes region-specific gene expression in the cortex. (a, d, g, j) Dorsal views of P1 brains processed for whole mount *in situ *analysis with digoxigenin labeled-cad8 riboprobes following electroporation at an anterior (a, d), or posterior (g, j) location of empty control (a, g) or Sp8 (d, j) vectors at E11.5. Arrowheads indicate the posterior border of the cad8 expression domain in anterior cortex. Anterior electroporation of Sp8 results in an anterior contraction of the cad8 domain (d), whereas posterior electroporation of Sp8 results in a posterior expansion of the cad8 domain (j). Electroporation of an empty vector has no effect on the cad8 domain (e, m). (b-f, h-l) Sagittal sections of P1 brains stained with an anterior marker gene, *RORb *probes (b, e, h, k), or a S1 marker gene, ephrin-A5 probes (c, f, I, l), following electroporation at an anterior (b, c, e, f) or posterior (h, I, k, l) domain of empty vector (b, c, h, i) or Sp8 (e, f, k, l). Arrowheads indicate the posterior border of the *RORb *expression domain or the putative M1/S1 and S1/V1 borders of ephrin-A5 expression. Coincident to the area shifts detected by cad8 expression, RORb and ephrin-A5 expressions are shifted anteriorly in anterior Sp8-overexpressed brains, and shifted posteriorly in posterior Sp8-electoporated brains. Scale bars: 0.5 mm (e, f, m, l); 0.5 mm (b, c, e, f, h, I, k, l). (m) Quantification of cad8 expression domains in *Sp8*- or control transfected brains. Anterior electroporation of *Sp8 *reduces motor area (M_A_) size detected as cad8 domain (8.2 ± 1.12%, SEM, n = 13) compared to the control cases (15.2 ± 0.297%, SEM, n = 9). In contrast, posterior electroporation of *Sp8 *enlarges motor areas (19.97 ± 0.771%, SEM, n = 6) compared to the control cases (14.37 ± 0.241). The bottom panel shows a scatter plot of individual cases. EP, electroporation.

Compared to cases electroporated with a control vector (n = 9 of 9, Figure 10a), we observed that in cases with anterior electroporations of *Sp8*, the anterior (motor) cad8 expression domain exhibits an anterior contraction (n = 7 of 11, Figure 10d). In contrast, cases with a posterior electroporation of *Sp8 *results in a posterior expansion of the anterior *cad8 *domain (n = 5 of 6, Figure 10j) compared to control cases (n = 6 of 6, Figure 10g). In addition, the expression patterns of *RORβ *(n = 4 of 4, Figure 10e) and *ephrin-A5 *(n = 4 of 4, Figure 10f) are shifted anteriorly in the anterior *Sp8 *transfected cases compared to the control transfected cases (n = 4 of 4, Figure 10b, c), or shifted posteriorly in the posterior Sp8 transfected cases (n = 4 of 4, Figure 10k, l) compared to control transfected cases (n = 4 of 4, Figure 10h, i). Thus, these findings indicate that ectopic *Sp8 *expression induces shifts in primary sensory areas revealed by serotonin immunostaining at P7, which is preceded by changes in the expression patterns of gene markers indicative of the positional identities of cortical neurons.

In summary, although *Sp8 *can strongly induce *Fgf8 *and its signaling pathway, the changes in area patterning and the parallel shifts in positional identity of cortical neurons induced by ectopic expression of *Sp8 *oppose those induced by ectopic expression of *Fgf8*. These unexpected findings suggest the complexity of the role for Sp8 signaling in influencing cortical patterning. *Sp8 *might interfere with or counteract the influence of *Fgf8 *on area patterning without disturbing *Fgf8 *signaling. These opposing effects of *Sp8 *and *Fgf8 *may cooperate to balance A-P patterning of the cortex into areas.

## Discussion

### Expression of the *Sp8*-like family relates to forebrain patterning centers

**Figure 11:**
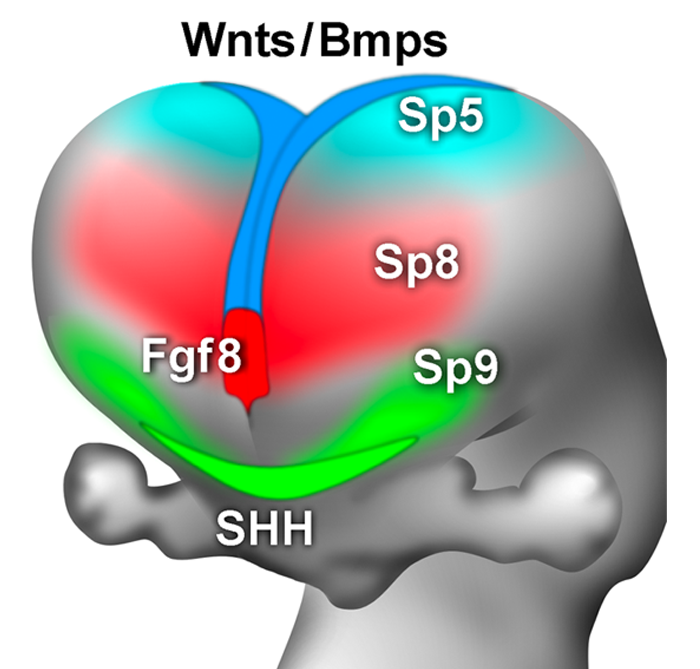
Summary of the *Sp8-*like family expression patterns related to telencephalic patterning centers. Position of patterning centers in relationship to Sp expression at E10.5. *Sp5 *expression is observed around the cortical hem, which expresses *Wnts *and *Bmps*, as being highest in medial and posterior parts of dTel. *Sp8 *is expressed in a high to low anterior-medial to posterior-lateral gradient across the entire cortical ventricular zone, and transiently overlaps with *Fgf8 *expression in the commissural plate. *Sp9 *is highly expressed in the mantle zone of the MGE, coincident with the domain of *Shh *expression. See the text for details.

We show that three mammalian homologues of the *Drosophila btd *gene, *Sp5*, *Sp8 *and *Sp9*, which are functionally most closely related to *btd*, are expressed in the embryonic mouse forebrain in unique patterns that closely relate to three signaling centers defined by the expression domains of distinct classes of morphogens implicated in forebrain patterning. *Sp5 *is expressed in association with the cortical hem and CPe, which are sources of *wnt*s and *Bmp*s, *Sp8 *with the ANR/CoP, a source of *Fgf*s, including *Fgf8*, and *Sp9 *with the *Shh *domain in ventral telencephalon (Figure 11). In addition, *Sp8 *and *Sp5 *are expressed within the cortical VZ in opposing gradients across the A-P axis of the cortex, with Sp8 being expressed in a high to low A-P gradient, whereas both are expressed in a high medial to low lateral gradient. These unique expression patterns of *Sp5*, *Sp8*, and *Sp9 *and their associations with forebrain signaling centers implicate them in forebrain patterning, potentially through their regulation of the relevant morphogens and their signaling.

Based on these expression patterns, we focused on a functional relationship between *Sp8 *and *Fgf8*, as well as potential roles for *Sp8 *in area patterning of the neocortex. It is not possible to study area patterning using constitutive *Sp8 *knockout mice because they exhibit an early embryonic lethality and have severe neural tube closure defects resulting in a failure of the cortex to develop [9,11]. Indeed, even the use of conditional knockout strategies to study roles for *Sp8 *in regulating *Fgf8 *independent from controlling cortical area patterning would be difficult as it would require the use of distinct Cre lines in which Cre recombinase is expressed in very restricted patterns, both spatially and temporally, to dissociate the function of *Sp8 *in regulating *Fgf8 *versus a direct role in cortical progenitor cells, and the requirement of *Sp8 *for neural tube closure and subsequent development of the cerebral cortex. To circumvent these obstacles, we used complementary gain of function and loss of function strategies employed through *in utero *electroporation of expression constructs, a technique similar to that recently used by others to show roles for *Fgf8 *in area patterning [18,19].

### *Sp8 *and *Fgf8 *share a reciprocal induction loop that exhibits significant specificity

We analyzed the relationship between *Fgf8 *and *Sp8 in vivo *by electroporating expression constructs into the lateral wall of the telencephalon and analyzing their influence at embryonic stages very early in cortical development, when *Fgf8 *is normally expressed in the CoP and exerts an influence on area patterning. We show that ectopic expression of *Fgf8 *in the telencephalon strongly induces *Sp8*. This finding is consistent with the recent observation that *Sp8 *expression is diminished in conditional or hypomorphic *Fgf8 *knockout mice [27].

We also found that ectopic expression of a full length *Sp8 *construct can strongly induce Fgf8 in dTel. To extend these findings, we show that ectopic expression in dTel of a 'dominant-active' *Sp8-VP16 *construct, created by the fusion of *Sp8 *with the transcriptional activator *VP16*, also robustly induces *Fgf8*, similar to the full length of *Sp8*, indicating that *Sp8 *is a transcriptional activator for *Fgf8*. We complemented these *in vivo *studies with an *in vitro *luciferase assay in transfected cell lines, and show that *Sp8 *strongly induces expression of an *Fgf8 *reporter construct *in vitro*. Thus *Sp8 *appears to directly induce *Fgf8*.

The reciprocal inductive loop between *Sp8 *and *Fgf8 *appears to be a specific relationship, consistent with the unique relationship in their expression patterns in the developing forebrain. The inductive effect of *Fgf8 *in the telencephalon is specific for *Sp8*, because we found that *Fgf8 *does not induce either *Sp5 *or *Sp9 in vivo*. Complementing these findings, we show using *in utero *electroporation in dTel that *in vivo Sp9 *only weakly induces *Fgf8*, and *Sp5 *has no detectable inductive effect. These findings are paralleled by those from the *in vitro *luciferase assay in which *Sp9 *weakly induced expression of an *Fgf8 *reporter construct. Interestingly, the inductive abilities of these *Sp*s for *Fgf8 *correlate with sequence homologies: Sp9 has identical *btd *and Zn finger domains to those of Sp8, whereas those of Sp5 are distinct [8].

In addition, the *Sp8/Fgf8 *inductive loop has specificity within the *Fgf *family, as indicated by our finding that *Sp8 *does not induce *Fgf17*, which is closely related to *Fgf8 *and is also expressed within the CoP. Thus, the reciprocal induction between *Sp8 *and *Fgf8 *exhibits significant specificity for one another. Such unique pairings of reciprocal induction between Sp8-like family members and forebrain patterning centers may also be utilized by *Sp9 *and Shh in ventral telencephalon and by *Sp5 *with wnts and Bmps in the cortical hem. This latter possibility is consistent with the report that the zebrafish homologue of *Sp5 *is induced by Wnt/β-catenin signaling [36].

### Distinct roles for *Sp8 *in initiation and maintenance of *Fgf8 *expression in the CoP

The initiation and maintenance of Fgf8 expression in the CoP are important phenomena for the regulation of cortical area patterning. Our findings show that ectopic *Sp8 *expression is sufficient to induce *Fgf8*, but *Sp8 *does not appear to be required for the normal initiation of *Fgf8 *expression in the ANR/CoP. Analysis of *Sp8 *knockout mice show that *Fgf8 *expression is initiated in the ANR, although its subsequent expression in the ANR, or later in the CoP, is not described [11]. Our findings, however, that *Fgf8 *expression is diminished when we electroporate a dominant-negative Sp8-engrailed construct into the CoP indicate that *Sp8 *is required to maintain *Fgf8 *expression in the CoP. This interpretation is also consistent with our expression data revealing that *Fgf8 *expression in the CoP diminishes at a stage shortly after *Sp8 *expression is no longer detected in the CoP but is enhanced outside of it within the cortical VZ. Thus, we conclude that although *Sp8 *is sufficient to induce *Fgf8*, it is not normally required for *Fgf8 *induction in the ANR/CoP. However, *Sp8 *is required for the maintenance of *Fgf8 *expression in the CoP. This scenario is similar to conclusions derived from observations of limb development in *Sp8 *knockout mice that *Fgf8 *expression is initiated but not maintained [11].

### *Sp8 *induction of *Fgf8 *is limited to the CoP by Emx2 repression

A related important issue is the mechanism by which *Fgf8 *expression is limited to the CoP even though *Sp8*, which can robustly induce *Fgf8 *in the telencephalon outside of the CoP, is more broadly expressed in dTel. One possibility is that other factors uniquely present in the CoP are required to cooperate with Sp8 to induce *Fgf8*. Another possibility, and not mutually exclusive, is that a repressor of Sp8 induction of *Fgf8 *is present in the dTel but not the CoP. Multiple lines of evidence indicate that this repressor is Emx2, a homeodomain transcription factor that controls area patterning.

**Figure 12:**
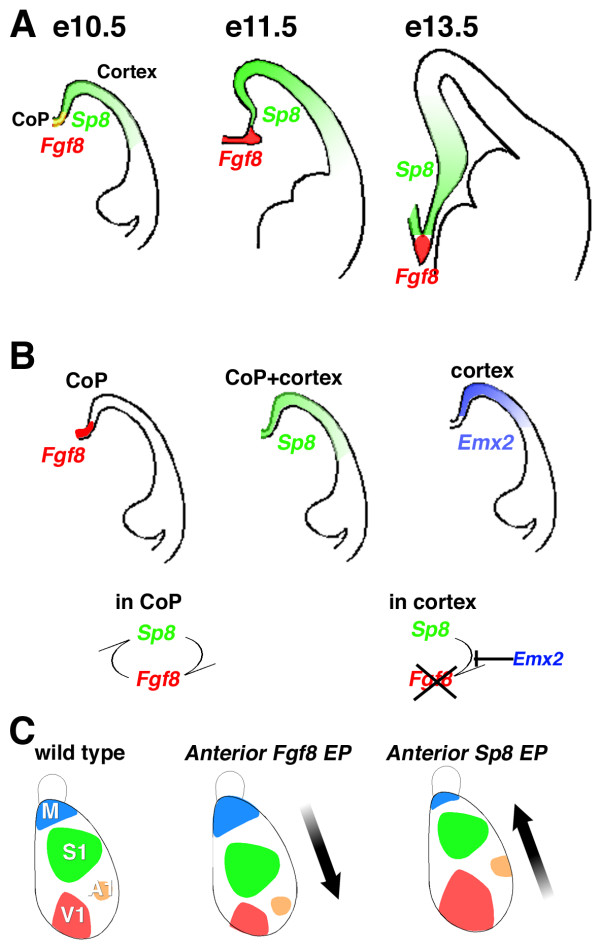
Summary of the Sp8 function related to telencephalic patterning centers and cortical area patterning. **(a) **Schematic diagram of time-dependent expression domains of Sp8 and Fgf8. At E10.5, Sp8 expression (green) is expressed in both progenitor cells in the cortical VZ (cortex) and within the CoP, where it overlaps with the *Fgf8 *expression domain (overlapping Sp8 and Fgf8 expression in the CoP is colored yellow). Sp8 expression is gradually excluded from the Fgf8 expression domain (red) at E11.5 and later. **(b) **Domain-dependent regulation of Sp8 TF activity by Emx2. Sp8 forms a reciprocal induction loop with Fgf8 in the CoP. Emx2 (blue) is expressed in cortical progenitors, but not in the CoP. Although Sp8 is expressed in cortex, Emx2 represses the ability of Sp8 to induce Fgf8, thereby restricting Fgf8 expression to the CoP. **(c) **Electroporation (EP) of *Fgf8 *and *Sp8 *results in opposing shifts in cortical area patterning. In early stages of cortical patterning, Sp8 maintains Fgf8 expression in CoP and the Fgf8 signaling pathway, which imposes anterior identity to cortical progenitors. Anterior EP of Fgf8 expression constructs results in enhanced anterior area identities and a corresponding posterior shift in cortical areas. Anterior EP of Sp8 expression constructs has an opposing effect on area patterning to that of Fgf8, and results in an anterior shift in cortical areas. Posterior EP of either Fgf8 or Sp8 have the opposing effect on area patterning compared to their anterior EP. These opposing effects of ectopic expression of Sp8 on area patterning compared to Fgf8 indicate that Sp8 activates a signaling pathway(s) that can overcome the effect of Fgf8 signaling, perhaps by interfering with Fgf8 signaling or dominating it. Furthermore, Sp8 expression in cortical progenitors may trigger distinct signaling pathways that function to facilitate posterior area identity.

First, we show using the *in vitro *luciferase assay that Emx2 completely represses the robust induction of *Fgf8 *by Sp8. In addition, the expression patterns of *Sp8 *and *Emx2 *are consistent with this model. At early stages, *Sp8 *expression overlaps with the CoP, and later becomes excluded from the CoP and spreads across the entire cortical VZ (Figure 12), whereas, *Emx2 *is never expressed in the CoP but is coincident with the expression of *Sp8 *in the cortical VZ [20,33]. Further, in *Drosophila*, homologues of vertebrate *Emx2 *and *Sp8*, *ems *and *btd*, respectively, interact to influence head development [37]. Taken together, these findings provide a compelling case that Emx2 represses Sp8 induction of *Fgf8 *in dTel and restricts *Fgf8 *expression to the CoP.

This interpretation is consistent with the report that the domain of *Fgf8 *expression expands in dTel beyond the CoP in *Emx2 *mutants [19]. Furthermore, our findings provide an explanation for the report that ectopic expression of *Emx2 *constructs electroporated into the CoP of forebrain slices can repress *Fgf8 *expression when done at E11.5 embryonic stages but not at E13.5 [19]. This temporal change in the ability of *Emx2 *ectopically expressed in the CoP to repress *Fgf8 *expression coincides with the early presence then downregulation of Sp8 expression in the CoP (present study). In conclusion, our findings indicate that the report that Emx2 both limits Fgf8 expression to the CoP and represses *Fgf8 *in the CoP by Emx2 [19] are due to Emx2 repression of Sp8 induction of Fgf8 (Figure 12).

### Ectopic *Sp8 *expression affects cortical area patterning in an opposing manner to *Fgf8*

We show that ectopic expression of *Sp8 *in either anterior or posterior dTel at embryonic stages early in cortical development has a strong effect on cortical area patterning, both the size and positioning of areas defined by serotonin staining, as well as the expression patterns of areal markers. The timing of our electroporations, and the postnatal stages of our analyses of area patterning are similar to those used by Fukuchi-Shimogori and Grove [18,19] to study the role of *Fgf8 *in area patterning. They showed that anterior ectopic expression of *Fgf8 *causes posterior area shifts, a finding that we have confirmed. Because anterior ectopic expression of *Sp8 *induces *Fgf8*, we expected that anterior ectopic expression of *Sp8 *would exhibit the same phenotype as anterior ectopic expression of *Fgf8*. Surprisingly, though, we found that anterior ectopic expression of *Sp8 *results in an anterior shift of areas, the opposite effect reported for *Fgf8*. We also found that posterior ectopic expression of *Fgf8 *and *Sp8 *have opposing effects on area patterning: posterior ectopic expression of *Sp8 *results in a posterior shift of areas, whereas *Fgf8 *results in an opposing anterior shift. Since the same ectopic expression of *Sp8 *not only induces *Fgf8*, but also induces TFs of the ETS family that are downstream factors of *Fgf8 *signaling, including *Pea3*, *Erm *and *Er81*, the opposing effects of ectopic expression of *Sp8 *versus *Fgf8 *on area patterning is unlikely due to *Sp8 *triggering an aborted or defective *Fgf8 *signal cascade.

Thus, we hypothesize that *Sp8 *can induce a signaling pathway that influences area patterning in a manner that opposes the *Fgf8 *pathway and competes with or balances the effect of Fgf8 signaling on area patterning. Fgf8 signaling might be balanced delicately, so that small changes of Sp8 activity might result in opposing outputs; consistent with this suggestion are the findings that different dosages of Fgf8 result in opposite effects on cell death/survival and forebrain patterning [27,38]. Sp8 might have dual roles in area patterning. In addition to a role in inducing *Fgf8*, *Sp8 *could have a more direct influence on the specification of the area identity of cortical progenitor cells that express it, such as that described for the TF EMX2 [20-22,33]. Indeed, an intriguing study in *Drosophila *shows that *ems*, the homolog of vertebrate *Emx2*, specifies the identity of the head segment, but only when acting with *btd*, the functional homolog of vertebrate *Sp8 *[37]. Thus, *Sp8 *may specify area identities in progenitors that express both it and *Emx2*, in opposing A-P gradients, potentially through an interaction with *Emx2*.

## Methods

### Animals

Timed pregnant ICR mice were used in accordance with Institution guidelines. ROSA26 reporter mice were obtained from Jackson Laboratories, and established homozygous males were crossed with ICR females to obtain heterozygous embryos. The day of insemination and the day of birth are designated as embryonic day 0.5 (E0.5) and postnatal day 0 (P0), respectively.

### *In situ *hybridization

The following S^35^- or digoxigenin (DIG)-labeled riboprobes were used: *Sp5*, *Sp8*, *Sp9*, *Fgf8 *[33], *Wnt2b *(image clone 353765), *Shh *[39], *Pea3*, *Er81 *(RT-PCR), *Erm *(image clone 5033778), ephrin-A5, RORβ [21]. *In situ *hybridization was done on 20 μm cryostat sections and DIG whole mount *in situ *hybridization was performed as described previously [34].

### *In utero *electroporation and expression constructs

Expression constructs were made by fusing cDNA of chicken *Fgf8 *isoform b, full length *Sp8*, *Sp8-VP16*, *Sp8-engrailed Sp5 *or *Sp9*, which was then subcloned into a Bluescript SK(-) vector containing chicken β-actin promoter from pCAGGS vector and a polyA sequence of bovine growth hormone sequences.

Surgery and *in utero *electroporation were performed according to the method by Saito *et al*. [40] with some modifications as follows. Plasmid DNA (1 mg/ml for genes of interest and 1 mg/ml for pCAG-nlsEGFP or pCAG-nls-cre), mixed with 0.1% Fast green for visualization, was injected with a glass capillary at E11.5 into the lateral ventricle to transfect cells in the VZ of dTel. One of two alternative methods were then followed: paddle-type electrodes (Nepagene CUY21, 0.5 cm diameter, 30 V, 50 mS × 5) were used to obtain broad electroporation sites for studies of gene induction and needle type electrodes made of tungsten were used for area analysis to ensure precise control of the electroporation site (80 V, 50 mS × 3). Embryos were later harvested, fixed and processed. The survival rate of embryos was approximately 70%. Approximately 77% exhibited efficient GFP expression (n = 105/137) when analyzed at E13 and E14. Targeting accuracy of the electroporation sites was also evaluated by the site of the GFP domain at E13 or E14: accuracy was 76% for anterior injections (n = 87/115) and 82% for posterior injections (n = 18/22). Only one case of anterior injection resulted in middle expression of the A-P axis, and the rest were negative for GFP signals.

### *In vitro *promoter assay

A 585 bp fragment, containing a 555 bp region immediately upstream of the mouse *Fgf8 *transcription start site and 30 bp of 5'-UTR of *Fgf8*, was subcloned into a promoter-less pGL3 basic vector (a firefly luciferase reporter vector; Promega, Madison, WI, USA), and transfected into C3H10T1/2 cells. Luciferase activity was measured after a 48 h transfection period as described previously [29].

### Gel retardation assay

Full length *Sp8 *was subcloned into pcDNA3.1 (Invitrogen, Carlsbad, CA, USA) and incubated in reticulocyte lysates following the manufacture's protocols (Promega) for *in vitro *translation. For the gel retardation assay, 5 μl of lysate was incubated with 1 ng of the P^32^-labeled 585 bp fragment of DNA corresponding to the *Fgf8 *promoter region in binding buffer (20 mM Hepes-KOH, pH 8.0, 5 μg/ml poly(dI-dC), 2% polyvinyl alcohol) for 1 h on ice in the presence or absence of unlabeled fragments as indicated in the figure 6b. Binding products were separated in 4% polyacrylamide gels and analyzed by phosphoimage analyzer (Typhoon 8600, Molecular Dynamics, Sunnyvale, CA, USA).

### Pull-down assay using biotinylated oligonucleotides

Biotinylated oligonucleotides containing the putative Sp8 binding site in the *Fgf8 *promoter (-230 to -190 from the transcription start site) were used. For control experiments, we introduced a mutation into the core region of the Sp1-recognition sequence, replacing GCC with TTT (designated as lower case t in the sequence below), based on a previous study [28]. The sequences of the wild-type (WT) and mutated oligos (Mut) were: WT, 5' TGCCCGCCGCCCCGCCCCCGGCCGCCCCGCCCCCGCCCCG; Mut: 5', TGCCCGCCtttCCtttCCCGGCCtttCCtttCCCtttCCG.

A pair of complementary oligonucleotides was annealed and used as a probe. C3H10T1/2 cells were transfected with pCAGGS or pCAGGS-HA-tagged *Sp8 *with Fugene6 (Roche, Indianapolis, IN, USA) according to the manufacturer's instruction. Cell lysates were harvested 48 h after transfection and each cell lysate was split in half to react with WT and Mut oligos. A biotin-streptavidin pull down assay was performed for the analysis of Sp8 binding to probes as previously described [41,42].

### Histochemistry and quantification of area size

Serotonin immunostaining and X-gal staining of tangential sections were performed as described previously [33]. The ratio of the length of motor areas and total areas of serotonin immunostained flattened brains, or cad8 expression domains of whole mount *in situ *P2 brains as shown in schematic diagrams in each figure, were measured by Image J software [43].

## Competing interests

The author(s) declare that they have no competing interests.

## Authors' contributions

SS conceived of the studies, engineered most of the expression constructs, designed and carried out most of the experimental studies, and helped draft the paper. YK performed the luciferase assays and pull-down assays, and engineered some of the expression constructs. JCIB provided unique reagents and oversaw the assays done by YK. DDMO helped conceive of the studies, coordinated them, analyzed and interpreted data, and drafted the paper. All authors read and approved the final manuscript.

## Supplementary Material

Additional File 1 Bmp signaling is not involved in Sp8-mediated Fgf8 upregulation. It is possible that Sp8-mediated Fgf8 upregulation could be due to a suppression of Bmp signaling, because Fukuchi et al. [30] reported that Noggin, a Bmp inhibitor, induces Fgf8 expression in vivo when overexpressed in forebrain. We thus tested whether manipulating Bmp activity can mediate Sp8 transcriptional activation of Fgf8 (see Legend to Additional file 1 below). First, we tested if C3H10T1/2 cells in our assay respond to manipulation of Bmp signaling. For this purpose, we used a Id1-reporter construct that has been shown to be activated by Smad1-dependent manner [41,44] In a condition of culture containing 1% FBS, we detected endogenous Bmp-mediated Id1 reporter activation compared to a deletion construct lacking Smad binding sites. Addition of Noggin reduced the reporter activity to the similar level of mutant reporter, indicating that basal activity of the reporter is due to Bmp-like activity in the 1% FBS and Noggin effectively blocks extrinsic Bmp activity in this system. In addition, co-transfection of constitutively active BMP receptors (CA-Alk3), also induced Id1 reporter activation, that was not blocked by an intracellular antagonist Noggin. Addition of Bmp2 protein, in a dose-dependent manner, stimulated the reporter activity. We thus conclude that C3H10T1/2 cells respond to Bmp signaling. We next tested whether Noggin elicits the expression of the Fgf8 reporter construct in C3H10T1/2 cells. We hypothesized if Sp8 induction of Fgf8 is mediated by the inhibition of Bmp pathways, such as an induction of Noggin as shown by Fukuchi et al in vivo, we expect that Sp8-mediated Fgf8 reporter expression will be affected by the presence of Noggin or Bmp proteins. However, we did not observed a significant upregulation of Fgf8 reporter expression in the presence of Noggin, though it slightly upregulates it (see error bar). If this minor change by Noggin reflects synergistic action of the inhibition of Bmp signaling and Sp8-mediated Fgf8 regulation, we would see that oppose effect of Bmp2 to the addition of to Noggin, i.e., suppression of Sp8 induction of Fgf8 reporter expression by Bmp. However, again, we did not see any significant downregulation of reporter expression by Bmp2 presence. Thus we conclude that altered Bmp signaling in our in vitro reporter assay does not mediate Sp8-mediated Fgf8 upregulation. In support of our conclusion, it was previously shown that Fgf8 upregulation by Noggin is observed when Noggin is overexpressed at E9.5, but not later than this time point [30]. In contrast, we detect Fgf8 upregulation by Sp8 when Sp8 expression construct is introduced at E11.5, the time point that overexpression of Noggin no longer upregulate Fgf8 in forebrain. So we propose that the Bmp signaling restricts Fgf8 expression in early stages, but not in later stages when still Sp8 regulates Fgf8 expression. (A) C3H10T1/2 cells respond to Bmp signaling. C3H10T1/2 cells were transfected with reporter construct of Id1 promoter (Id1-Luc) or mutated construct lacked Smad binding sites that does not respond to either serum in culture medium or Bmp2 protein (100 ng/ml). Transfection of Alk3, constitutively active BMP receptors (CA-Alk3), induces Id1 reporter expression in a Smad-dependent manner. Addition of Noggin (100 ng/ml) inhibits endogenous Bmp signaling. Bmp2 can elicit Id1 reporter expression in a dose dependent manner and saturated at 10 ng/ml. (B) Sp8-mediated Fgf8 upregulation is independent from Bmp signaling. The expression of Fgf8 reporter construct mediated by Sp8, or endogenous factors, is not significantly affected in the presence or the absence of Noggin, or Bmp2 proteins. Click here for file

Additional File 2 Tangential sections of ROSA26 reporter mouse brains electroporated with a *CAG-VP16 *control expression vector (A, C) or a dominant active *CAG-Sp8-VP16 *expression vector (B, D) together with a *CAG-Cre *expression vector. Counterstaining of LacZ in sections indicates the position of the cells electroporated with a nuclear-localized-signal (nls)-Cre. Direct detection of GFP signal shows that approximately 80% of cases electroporated at E11.5 and analyzed at E13.5 have accurately positioned electroporations. However, because the eGFP reporter co-electroporated at E11.5 with the *Sp8 *constructs is not detectable at P7, to confirm these results, we repeated these studies using ROSA26 reporter mice by co-electroporating a CAG-Cre Recombinase construct with the *Sp8-VP16 *dominant active construct and the *VP16 *control construct. The findings confirmed that in every case (n = 4 of 4), the transfection domains, marked by X-gal labeled cells, is at either the targeted cortical pole (anterior or posterior), and the area shifts are identical to those described above. (see Methods for details). Scale bar: 1.0 mm. Click here for file
