# Supplementary figures and images for: Sp8 exhibits reciprocal induction with Fgf8 but has an opposing effect on anterior-posterior cortical area patterning
Source: Neural Dev. 2007 May 17;2:10. doi: 10.1186/1749-8104-2-10 (PMC1890288; doi:10.1186/1749-8104-2-10)

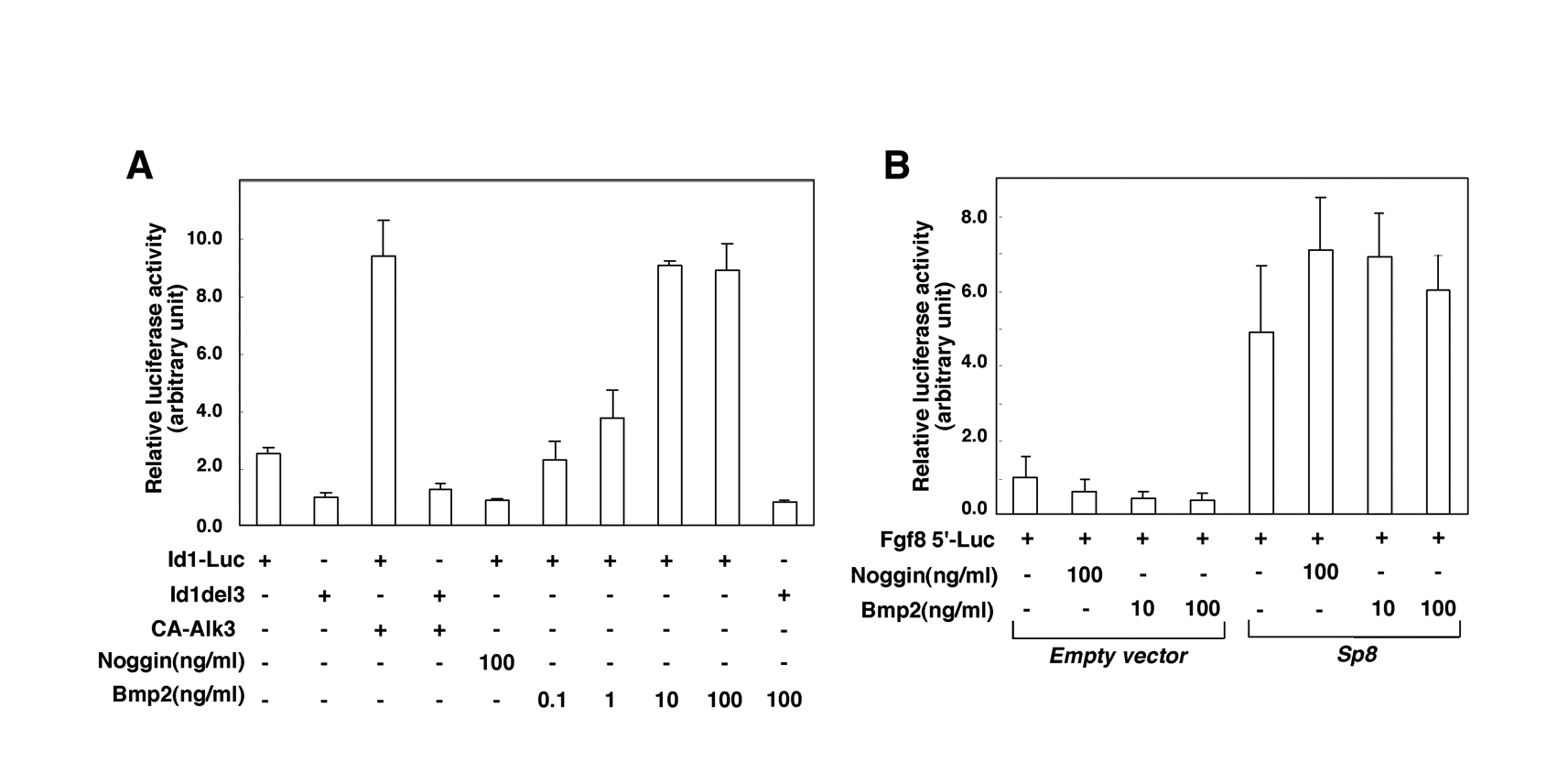

Supplement: Additional File 1 — Bmp signaling is not involved in Sp8-mediated Fgf8 upregulation. It is possible that Sp8-mediated Fgf8 upregulation could be due to a suppression of Bmp signaling, because Fukuchi et al. [30] reported that Noggin, a Bmp inhibitor, induces Fgf8 expression in vivo when overexpressed in forebrain. We thus tested whether manipulating Bmp activity can mediate Sp8 transcriptional activation of Fgf8 (see Legend to Additional file 1 below). First, we tested if C3H10T1/2 cells in our assay respond to manipulation of Bmp signaling. For this purpose, we used a Id1-reporter construct that has been shown to be activated by Smad1-dependent manner [41,44] In a condition of culture containing 1% FBS, we detected endogenous Bmp-mediated Id1 reporter activation compared to a deletion construct lacking Smad binding sites. Addition of Noggin reduced the reporter activity to the similar level of mutant reporter, indicating that basal activity of the reporter is due to Bmp-like activity in the 1% FBS and Noggin effectively blocks extrinsic Bmp activity in this system. In addition, co-transfection of constitutively active BMP receptors (CA-Alk3), also induced Id1 reporter activation, that was not blocked by an intracellular antagonist Noggin. Addition of Bmp2 protein, in a dose-dependent manner, stimulated the reporter activity. We thus conclude that C3H10T1/2 cells respond to Bmp signaling. We next tested whether Noggin elicits the expression of the Fgf8 reporter construct in C3H10T1/2 cells. We hypothesized if Sp8 induction of Fgf8 is mediated by the inhibition of Bmp pathways, such as an induction of Noggin as shown by Fukuchi et al in vivo, we expect that Sp8-mediated Fgf8 reporter expression will be affected by the presence of Noggin or Bmp proteins. However, we did not observed a significant upregulation of Fgf8 reporter expression in the presence of Noggin, though it slightly upregulates it (see error bar). If this minor change by Noggin reflects synergistic action of the inhibitio [file 1749-8104-2-10-S1.tiff]

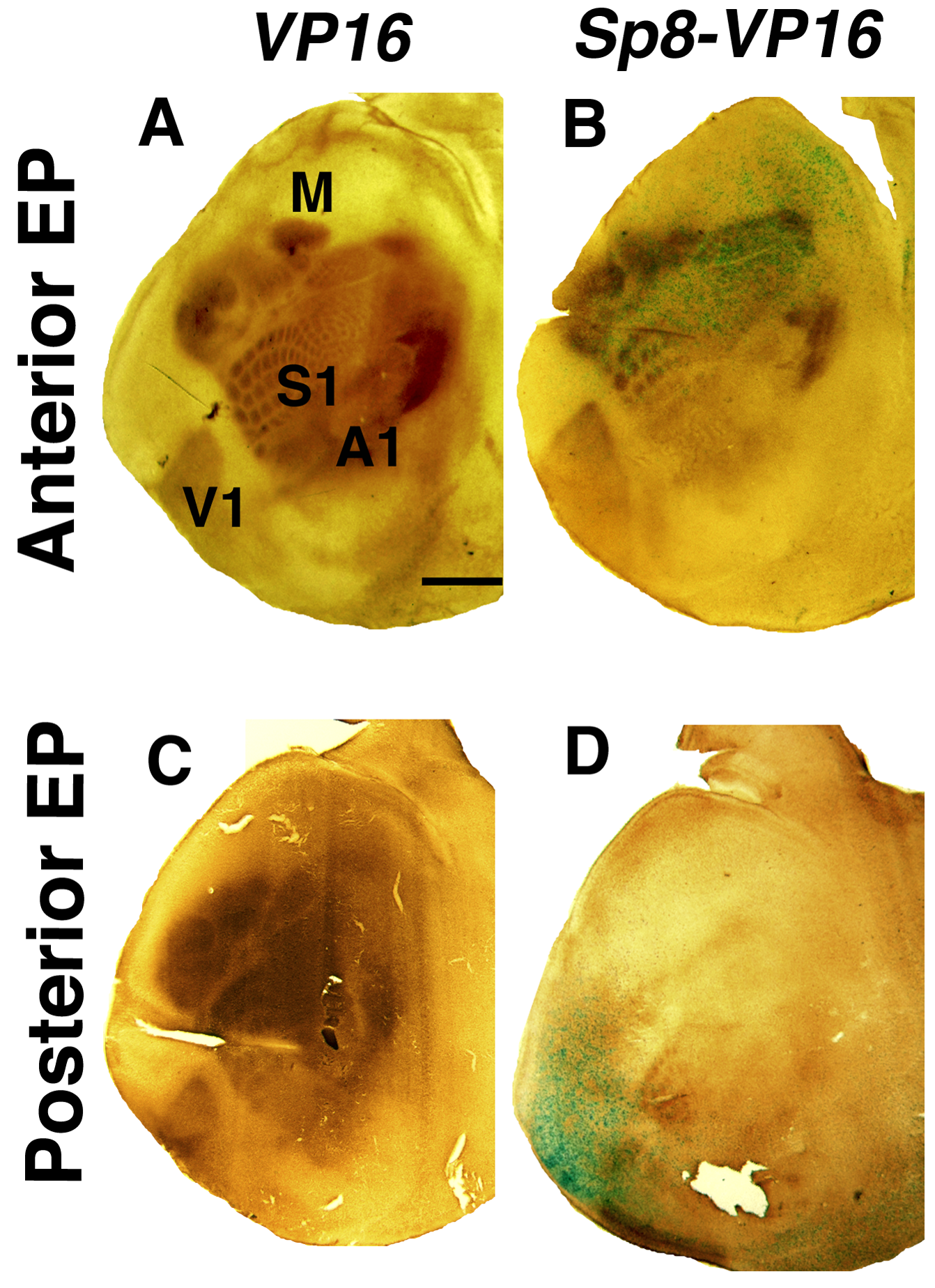

Supplement: Additional File 2 — Tangential sections of ROSA26 reporter mouse brains electroporated with a CAG-VP16 control expression vector (A, C) or a dominant active CAG-Sp8-VP16 expression vector (B, D) together with a CAG-Cre expression vector. Counterstaining of LacZ in sections indicates the position of the cells electroporated with a nuclear-localized-signal (nls)-Cre. Direct detection of GFP signal shows that approximately 80% of cases electroporated at E11.5 and analyzed at E13.5 have accurately positioned electroporations. However, because the eGFP reporter co-electroporated at E11.5 with the Sp8 constructs is not detectable at P7, to confirm these results, we repeated these studies using ROSA26 reporter mice by co-electroporating a CAG-Cre Recombinase construct with the Sp8-VP16 dominant active construct and the VP16 control construct. The findings confirmed that in every case (n = 4 of 4), the transfection domains, marked by X-gal labeled cells, is at either the targeted cortical pole (anterior or posterior), and the area shifts are identical to those described above. (see Methods for details). Scale bar: 1.0 mm. [file 1749-8104-2-10-S2.tiff]
